# Cardiovascular adverse events in chronic myeloid leukemia patients treated with nilotinib or imatinib: A systematic review, meta-analysis and integrative bioinformatics analysis

**DOI:** 10.3389/fcvm.2022.966182

**Published:** 2022-11-08

**Authors:** Sicong Li, Jinshan He, Xinyi Zhang, Yuchun Cai, Jian Liu, Xiaoyan Nie, Luwen Shi

**Affiliations:** ^1^School of Pharmaceutical Sciences, Peking University, Beijing, China; ^2^Department of Cardiovascular, Peking University People’s Hospital, Beijing, China

**Keywords:** chronic myeloid leukemia, nilotinib, imatinib, cardiovascular adverse events, atherosclerosis

## Abstract

**Objective:**

The aim of this article is to assess the risk and potential mechanisms of cardiovascular adverse events in patients treated with nilotinib or imatinib by conducting a systematic review, meta-analysis and integrative bioinformatics analysis.

**Materials and methods:**

Three databases were systematically searched for studies published from inception to May 29, 2022. Differential expression analysis and weighted gene coexpression network analysis (WGCNA) were performed to search for modules of genes most associated with cardiotoxicity. Protein-protein interaction (PPI) network analysis was then performed to identify hub genes for the cardiotoxicity of nilotinib. Molecular docking was used to analyze the effects of rosuvastatin and aspirin on these targets.

**Results:**

Patients treated with nilotinib as first-line treatment were associated with a higher risk of CAE (OR = 3.43 [95% CI 2.77–4.25]), CAD (OR = 5.30 [95% CI 3.85–7.29]), ACS (OR 2.7 [95% CI 1.60–4.54]), CVA (OR 5.76 [95% CI 2.84–11.28]), PAOD (OR 5.57 [95% CI 3.26–9.50]) and arrhythmia (OR 2.34 [1.17,4.67]) than those treated with imatinib, while no significant difference was found in the risk of HF (OR 1.40 [95% CI 0.42–4.69]) between the two groups. Patients who were treated with more than 600 mg daily dosage of nilotinib or followed up for more than 5 years had a higher risk of ACS and CVA. IL6, CXCL8, CCL2, SOD2, NFKBIA, and BIRC3 were identified as the top 6 hub genes in the magenta module (human cardiomyocyte samples) and were mainly enriched in the NOD-like receptor signaling pathway, IL-17 signaling pathway, TNF signaling pathway, lipid and atherosclerosis signaling pathway. TYROBP and CSF1R were identified as hub genes in the turquoise module (liver samples from Mus musculus). GSEA results showed that type II diabetes mellitus, B-cell receptor, apoptosis, insulin, natural killer cell mediated cytotoxicity,

mTOR, chemokine, and T-cell receptor signaling pathways were related to the higher risk of atherosclerosis caused by nilotinib. Rosuvastatin can effectively bind to most of the hub targets and proteins enriched in the inflammatory pathways above.

**Conclusion:**

CML patients who start with nilotinib have a higher risk of CAE than those with imatinib. Atherosclerosis caused by the inflammatory response and glycolipid metabolism disorder is the key mechanism of nilotinib cardiotoxicity. Rosuvastatin may be an effective treatment for the cardiotoxicity of nilotinib.

## Graphical Abstract

Study design for the bioinformatics analysis. 
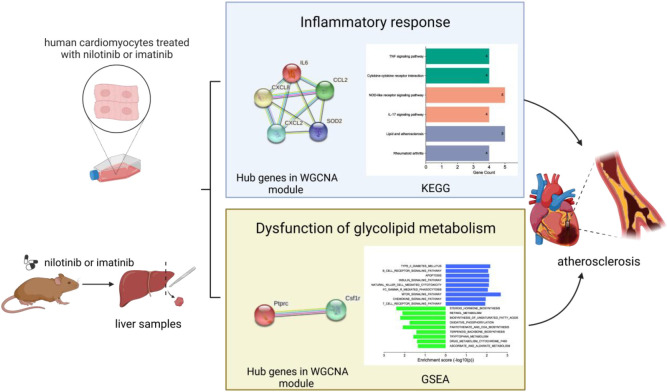

## Introduction

Chronic myeloid leukemia (CML) is a malignant hematopoietic system disease that severely endangers the life of patients. CML patients possess the Philadelphia chromosome, which contains the Bcr-Abl that encodes the oncoprotein BCR-ABL. As the first TKI approved by the FDA, imatinib can improve the outcomes of CML patients and prolong their overall survival to a point that is similar to their age-matched healthy individuals (1). However, inevitable drug resistance to imatinib and the majority of relapses upon withdrawal have occurred frequently due to several mutations in the BCR-ABL kinase. Effective against most BCR-ABL1 mutations (T315I excluded), nilotinib has been approved as a first-line treatment and second-line treatment for CML patients with intolerance or resistance to imatinib (2), with a 10- to 50-fold higher BCR/ABL kinase inhibition activity than imatinib (3). The clinical efficacy of nilotinib (300 mg BID, 400 mg BID) in newly diagnosed chronic phase CML was demonstrated in the randomized phase III ENESTnd trial (4). As reported in the phase II study GIMEMA CML 0307, the 10-year overall survival and progression-free survival in patients treated with nilotinib were 94.5% (5). The rates of major (MMR) and deep (MR4) molecular responses were 96% and 83%, respectively (5).

Apart from hematological, musculoskeletal, gastrointestinal and subcutaneous toxicity, nilotinib can also lead to adverse effects different from those of imatinib, such as cardiovascular adverse events (1, 6). A total of 23.3% of patients have at least one arterial obstructive event, which suggests that cardiovascular toxicity remains a concern. Nilotinib (NILO) can cause accelerated atherosclerosis and arterial thrombotic events (myocardial ischemia, stroke, and peripheral artery obstructive disease), hyperglycemia and hyperlipidemia (7, 8). The risk increases with the nilotinib administration duration (9). TKIs have become the current standard of care for CML, so their cardiotoxicity should be given enough attention in this population. The mechanisms underlying the cardiovascular adverse events induced by nilotinib or imatinib remain unclear.

Nowadays, statins have been recommended for optimal atherosclerotic cardiovascular disease (ASCVD) risk reduction by American College of Cardiology/American Heart Association (ACC/AHA) Guideline (10) and European Society of Cardiology/European Atherosclerosis Society (ESC/EAS) Guideline (11). As inhibitors of 3-hydroxy 3-methylglutaryl coenzyme A reductase, statins can reduce circulating low-density lipoprotein (LDL) and cholesterol levels by 25 to 50%. Moreover, statins bring about cardiovascular benefits via anti-inflammation and atherosclerotic plaque stabilization (12). As was reported in the Network Meta-Analyses conducted by Xiaodan Zhang et al. (13), rosuvastatin ranked first in lowering low-density lipoprotein cholesterol (LDL-C), apolipoprotein B (ApoB) and increasing apolipoprotein A1 (ApoA1) efficacy. Rosuvastatin, at moderate and high intensity doses, was the most effective in reducing levels of non-high density lipoprotein cholesterol in patients with diabetes (14). Therefore, we assess the therapeutic potential of rosuvastatin and aspirin, an important drug in prevention of ASCVD, so as to provide reference for researches on cardiotoxicity of nilotinib.

## Method

### Meta-analysis

#### Literature data sources and search strategy

This systematic review and meta-analysis were registered on the PROSPERO platform (CRD42022334398) and performed in accordance with the Preferred Reporting Items for Systematic Reviews and Meta-Analyses (PRISMA) guidelines (15). The Embase, PubMed, and Cochrane Library databases were searched for articles. Moreover, we searched https://www.isrctn.com, https://www.clinicaltrials.gov, and http://www.chictr.org.cn/index.aspx for registered trials. The retrieval time was from inception to May 29, 2022. The detailed search strategy is described in Supplementary Tables 1–3.

#### Inclusion and exclusion criteria

##### Inclusion criteria

(1) Chronic myeloid leukemia patients who started with nilotinib or imatinib as first-line treatment. (2) Sufficient data and full text available for meta-analysis. (3) Study types were randomized controlled trials (RCTs) or observational studies. (4) Articles published in English.

##### Exclusion criteria

(1) Patients treated with a TKI except for imatinib or nilotinib. (2) Rotation of imatinib and nilotinib during follow-up. (3) Study types were case reports, single-cell sequencing studies, animal experiments, conference presentations, study protocols, meta-analyses or network meta-analyses.

#### Definition of the outcome

Cardiovascular adverse events (CAE), which were defined as the combination of any of the following events (1) coronary artery disease (CAD), which included but not was limited to stable angina, or acute coronary syndrome(ACS) (including unstable angina, ST or non-ST segment elevation myocardial infarction) (2) cerebrovascular accident (CVA), including stroke or transient ischemic attack (TIA) (3) peripheral artery occlusive disease(PAOD) (4) heart failure(HF) (5) pulmonary hypertension (PH) (6) arrhythmia.

#### Study selection and data extraction

Two review authors (Sicong Li and Jinshan He) independently reviewed the titles and abstracts of studies with potential eligibility. After that, we downloaded the full texts of studies eligible for inclusion. Two authors (Xinyi Zhang and Yuchun Cai) independently extracted the following data: (1) basic information, including first author, publication year, sample size, follow-up time and study design; (2) characteristics of patients, including sex, age and country; (3) details about the TKI treatment: dosage and duration of nilotinib or imatinib treatment; and (4) information on quality assessment. Any disagreement concerning data extraction was settled through consensus among authors.

#### Strategy for meta-analysis

This meta-analysis was performed by using R (version 4.0.3). The chi-square test and I2 value were used to measure statistical heterogeneity. *I*^2^ < 50% and *P* > 0.05 indicated no significant heterogeneity, and a fixed-effects model was used to pool the value of OR/HR and 95% confidence interval; otherwise, a random-effects model was used. Subgroup analysis was conducted to analyze sources of heterogeneity. Sensitivity analysis was conducted by excluding one study each time. Begg’s and Egger’s tests were used to assess publication bias. Statistical significance was set as α = 0.05 in this study.

#### Quality assessment

XZ and YC assessed the quality of eligible studies independently by using the Newcastle–Ottawa Quality Assessment Scale (NOS) (16). The NOS assessed the quality of studies from the aspects of selection, comparability, and exposure, with a total score ranging from 0 to 9 points. More than 6 points was defined as a high-quality study. The results are presented in Supplementary Table 5.

### Bioinformatics analysis

#### Data acquisition and quality control

By using “Tyrosine kinase inhibitor,” “cardiotoxicity” and “atherosclerosis” as keywords, the Gene Expression Omnibus (GEO) repository^1^ was searched for datasets about the cardiotoxicity of nilotinib or imatinib. GSE146095 and GSE146096 with expression profiling of cardiomyocytes from Homo sapiens and GSE103908 with expression profiling of liver tissues from Mus musculus were obtained for further analysis (17). No vascular endothelial cell samples treated with TKI were found on the GEO website. We first used the inSilicoMerging package of R software to merge the two datasets (GSE146095 and GSE146096) (18). Then, we used the method illustrated by Johnson we et al. (19) to remove the batch effect and finally obtained the transcriptomic profile matrix of human heart-derived primary cardiomyocyte-like cell lines from 16 nilotinib samples and 20 imatinib samples.

The liver plays a central role in cholesterol metabolism and lipoprotein distribution. Moreover, the liver is the main organ for the degradation of insulin, which inhibits gluconeogenesis and promotes glycogen decomposition and the synthesis and metabolism of long-chain fatty acids and triglycerides.

In the study of GSE103908, histopathological analysis of atherosclerosis and transcriptome analysis of the liver were performed on female APOE*3Leiden CETP transgenic mice. Sixteen of them were treated with imatinib (150 mg/kg BID), and eight of them were treated with nilotinib (10 or 30 mg/kg QD). Baseline was defined as the time point after 3 weeks on a Western-type diet containing saturated fat from 15% (w/w) cacao butter and 0.15% cholesterol. Nilotinib decreased collagen content by 32% (*p* = 0.003 < 0.05) and the lesion stability index by 43% (*p* = 0.003 < 0.05). Increased expression of macrophage-derived chemokine monocyte chemoattractant protein-1 (MCP-1) was observed in the nilotinib group. Imatinib reduced average cholesterol and triglyceride levels by 69% (*p* < 0.001) and 36% (*p* = 0.019), respectively, which was related to inhibiting VLDL production and intestinal absorption of cholesterol (20).

#### Analysis of differentially expressed genes

First, the probe names were converted into gene symbol names. Second, DEGs were identified by using the “limma” package (adjusted *p* < 0.05 and | log2FoldChange | > 1). All of the DEGs were shown in a volcano plot, and the top 10 DEGs are shown in a heatmap.

#### Weighted gene co-expression analysis

The WGCNA package in R software was used to find clusters of highly correlated genes (with hierarchical clustering) and to summarize these clusters as module eigengenes (MEs) by liaising with cardiotoxicity and assigning module membership (MM) to genes. After obtaining the expression profile of differentially expressed genes, we removed the genes with a standard deviation of 0 in each sample, removed the outlier genes and samples by using the goodSamplesGenes method in the WGCNA package, and further constructed the scale-free coexpression network. Specifically, first, Pearson’s correlation matrices and the average linkage method were both performed for all pairwise genes. Then, a weighted adjacency matrix was constructed using the power function A_mn = | C_mn| ^β (C_mn = Pearson’s correlation between Gene_m and Gene_n; A_mn = adjacency between Gene m and Gene n). β was a soft-thresholding parameter that could emphasize strong correlations between genes and penalize weak correlations. After choosing the power of 20, the adjacency was transformed into a topological overlap matrix (TOM), which could measure the network connectivity of a gene defined as the sum of its adjacency with all other genes for network Gene ratio, and the corresponding dissimilarity (1-TOM) was calculated. To classify genes with similar expression profiles into gene modules, average linkage hierarchical clustering was conducted according to the TOM-based dissimilarity measure with a minimum size (gene group) of 30 for the gene dendrogram. Sensitivity was set as 2. To further analyze the module, we calculated the dissimilarity of module genes, chose a cut line for the module dendrogram and merged some modules. In addition, we also combined modules with a distance less than 0.25 and finally obtained four coexpression modules. Genes in the module most related to the cardiotoxicity of nilotinib were obtained for further analysis.

#### Gene ontology and kyoto encyclopedia of gene and genomes enrichment analysis

We used the DAVID website^2^ to perform GO function and KEGG pathway enrichment analyses for genes in the most relevant module (21). Each term was calculated with a P value by using Fisher’s exact test. *P* < 0.05 was considered statistically significant. All of the results were visualized by using the bioinformatic website.^3^

#### Construction and analysis of the protein-protein interaction network

The PPI network was constructed by using the STRING database^4^ with a confidence score > 0.4 (4). The downloaded results were imported into Cytoscape 3.8.2 (22) software for further analysis. The top 10 hub genes in the PPI network were screened out by using the cytoHubba plugin. UpsetR was used to take the intersection of the top 10 hub genes according to 5 criteria.

#### Construction of the competing endogenous RNA network

miRNA–mRNA and miRNA–lncRNA interactions were obtained by searching lncACTdb (23).^5^ In this database, we searched for ceRNA interactions supported by low- and high-throughput experiments. Finally, the ceRNA network was visualized in a Sankey plot by the R package “ggalluvial.”

#### Gene set enrichment analysis

We obtained GSEA software (version 3.0) from the GSEA website^6^ (24). We downloaded the c2.cp.kegg.v7.4.symbols.gmt subset from the Molecular Signatures Database^7^ (19) to evaluate the relevant pathways and molecular mechanisms based on gene expression profiles and phenotypic grouping. The default weighted enrichment method was used for the enrichment analysis. The random combination was set for 1000 times (25). | NES| > 1, FDR < 0.25, NOM *p* < 0.05 were considered significant enrichment.

#### Molecular docking

Molecular docking was performed to predict the binding of rosuvastatin and aspirin to the hub proteins and the targets enriched in the atherosclerosis signaling pathway. The three-dimensional structures of rosuvastatin and aspirin were obtained from the PubChem database,^8^ and the three-dimensional structures of hub proteins were obtained from the RCSB Protein Data Bank (PDB) database.^9^ Molecular docking simulations between rosuvastatin, aspirin and the target proteins were performed by using the AutoDock Tool (version 1.5.6) and AutoDock Vina 1.1.2 (Molecular Graphics Laboratory, Scripps Institute, 2011). A minimum binding energy less than 5 indicated a good binding ability. The results were finally visualized by using the PyMOL molecular graphics system (v.2.4.0, Schrödinger, LLC) (26).

## Results

### Results of meta-analysis

#### Literature search

**FIGURE 1:**
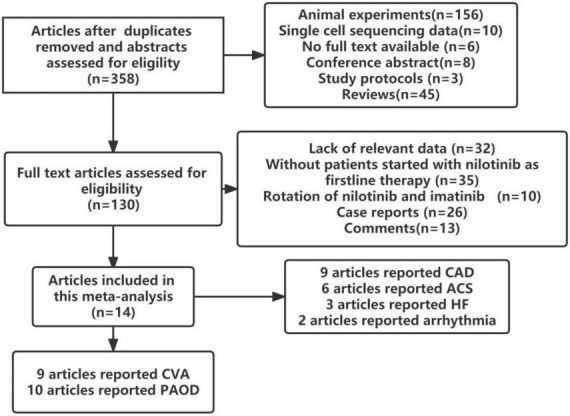
Flow chart of the study selection.

In total, 14 studies involving 9699 patients with CML were found to meet the inclusion criteria. Wang et al. (27) and Kantarjian et al. (28) reported open labeled randomized controlled studies, Anna Sicuranza et al. reported prospective cohort studies (29), while others reported retrospective cohort studies (30–40). The flow chart of the study selection process is presented in Figure 1.

#### Study characteristics

The included studies were published between 2013 and 2022 and were conducted in Italy, China, Sweden, Slovakia, the USA, Germany, Japan, and Ireland. The average follow-up time ranged from 4.2 years to 10 years. Six studies did not report the dosage of nilotinib and imatinib. The relevant characteristics of the included studies are detailed in Table 1. Pulmonary hypertension was not included in this analysis because only one study reported it.

**TABLE 1:** Basic characteristics of the included studies.

Author	Country	Sample size	Age (year-old)	Sex male(%)	CAE outcomes reported	Duration of nilotinib	Duration of imatinib	Nilotinib dose	Imatinib dose	Follow-up time
Subramanian et al. (24)	Japan	369	53.0 (range 18–89)	224 (60.70%)	CAD, CVA, PAOD	464 person-years	1336 person-years	150 mg QD,300 mg QD, 400 mg QD, 300 mg BID,	400 mg QD	71.8 (range 1–196) months
Zhao et al. (25)	Sweden	1601	imatinib 60 (range 46–70), nilotinib 60 (range 45–69)	715 (64%)	ACS,CAD,PAOD	2.8 (range0.8–5.6) years	3.2 (range1.1–7.8) years	NA	NA	6 (range 3–10) years
Trott and Olson (26)	Slovakia	82	55.82 ± 13	48 (58.54%)	CAD, CVA, PAOD	51.6 (range3.0–123.6) months	126.25 (range3.33–198.00) months	300 mg BID,400 mg BID	400 mg QD	median 61.3 months
Kantarjian et al. (27)	Sweden	896	58.2 ± 17.0	485 (54.1%)	ACS, CVA, POAD	167 person-years	2350 person-years	NA	NA	4.2 (range1.9–7.1) years
Wang et al. (28)	China	1,111	Nilotinib 48.3 ± 14.4; Imatinib 49.0 ± 16.4	NA	CAD,CVA,POAD	91.2 ± 277.6 days	35.8 ± 130.9 days	NA	NA	5 years
Sicuranza et al. (29)	USA	531	49 ± 15	321 (60%)	CAD, PAOD, HF, CVA, PH, Arrhythmia	77 (range 3–134) months	imatinib 400 mg cohort 144 (range, 2–195)months, imatinib 800 mg cohort 136 (2-186)months	400 mg BID	400 mg QD,400 mg BID	94 (range 2-196) months
Fujioka et al. (30)	Japan	506	56(range 18-92)	329(65%)	PAOD, ACS, HF, arrhythmia, CVA	65.3 (range2.0–89.2) months	77.9 (range 1.7–97.8) months	300 mg QD, 300 mg BID	300 mg QD	5 years
Dahlén et al. (31)	Ireland	1857	nilotinib median 47;imatinib median 49	1089(58.64%)	CAE	36 (range,0–47) months	45 (range 0–67) months	300 mg BID, 400 mg BID	400 mg QD, 400 mg BID	6 (IQR, 3-10) years
Petrikova et al. (32)	China	1207	46.38 ± 14.96	728 (60.31)	CAD, CVA, PAOD	median 2.40 years	median 3.74 years	NA	NA	NA
Szklarczyk et al. (4)	USA	846	NA	NA	CAD,CVA,PAOD	median 82.8 months in the 300-mg BID group, 87.5 months in the 400-mg BID group	median 64.0 months	300-mg BID, 400-mg BID	400 mg QD	10 years
Wang et al. (23)	Italy	186	60 (range 24–90)	(107/79)	ACS,CVA,PAOD	24 (range 12–64.5)months	21 (range 12–62.7) months	NA	NA	23.3 (range 12–64.6)months
Dahlén et al. (33)	Germany	159	53 (range 21–85)	(84/75)	PAOD	36 (range 6–72) months	97.5 (range 8–146)months	300 mg BID, 400 mg BID, 1200 mg QD, 600 mg BID	400 mg QD, 400 mg BID	74 (4–269) months
Ragueneau et al. (22)	China	267	nilotinib 41 (range 18–76), imatinib 39 (range 19–74)	172 (64.42%)	CVA	2 years	2 years	300 mg BID	400 mg QD	2 years
Chen et al. (34)	Italy	81	Median [IQR]:nilotinib 60 [53–66]; imatinib 62 [51–69]	43(53.09%)	ACS, HF, CVA	3.59 [IQR 2.23–4.76] years	4.55 [IQR 1.39–7.89] years	NA	NA	5.93 (IQR 3.64–9.25) years

#### Results for cardiovascular adverse events

**FIGURE 2:**
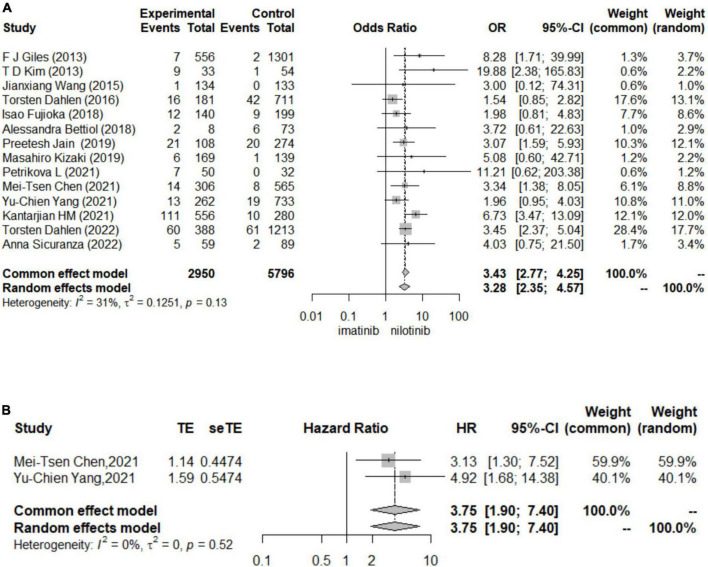
Forest plots for cardiovascular adverse events **(A)**. The results for logistic regression **(B)**. The results for survival analysis.

In logistic regression and survival analysis, patients treated with nilotinib as first-line treatment suffered from a higher risk of CAE (OR 3.43 [95% CI 2.77–4.25], HR = 3.75 [95% CI 1.90, 7.40]) than those treated with imatinib (see Figure 2). No individual study was found to significantly influence the pooled HR and 95% CI in the sensitivity analysis. No significant publication bias was found by Begg’s and Egger’s tests. Torsten Dahlén (33) contributed the most to the overall heterogeneity and the overall results. In the subgroup analysis, different definitions of CAE might be the main source of heterogeneity. In terms of survival analysis, we did not construct funnel plots or perform Begg’s test and Egger’s test to assess publication bias due to the less than recommended arbitrary minimum number of studies.

#### Results for other outcomes

**FIGURE 3:**
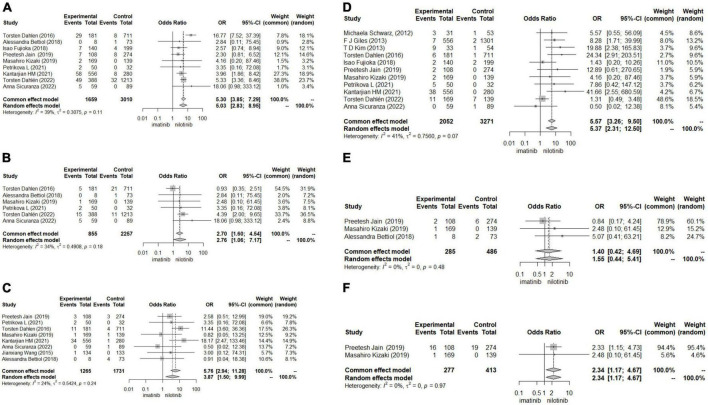
Forest plots for other outcomes. **(A)** Coronary artery disease (CAD). **(B)** Acute coronary syndrome (ACS). **(C)** Cerebrovascular accident (CVA). **(D)** Peripheral artery occlusive disease (POAD). **(E)** Heart failure (HF). **(F)** Arrhythmia.

Patients treated with nilotinib as first-line treatment had a higher risk of CAD (OR 5.30 [95% CI 3.85–7.29]), ACS (OR 2.7 [95% CI 1.60–4.54]), CVA (OR 5.76 [95% CI 2.84–11.28]), POAD (OR 5.57 [95% CI 3.26–9.50]) and arrhythmia (OR 2.34 [1.17,4.67]) than those treated with imatinib, while no significant difference was found in the risk of HF (OR 1.40 [95% CI 0.42–4.69]) between the two groups (Figure 3). The results of the publication bias assessment, sensitivity analysis and baujat plots for heterogeneity analysis are presented in Supplementary Figures 7–10.

Regarding the outcomes of HF and arrhythmia, we did not construct funnel plots or perform Begg’s test and Egger’s test to assess publication bias due to the less than recommended arbitrary minimum number of studies.

In subgroup analysis, sample size may be the source of heterogeneity in the comparison of ACS, CVA and CAD. Nilotinib treatment in studies with sample sizes greater than 1000 tended to show a higher risk of ACS, CVA and CAD than imatinib treatment. The median follow-up time, dosage and duration of nilotinib may be the source of heterogeneity in the comparison of ACS, which indicated that patients treated with more than 600 mg daily dosage or longer than 5 years of nilotinib treatment or who were followed up for more than 5 years suffered from a higher risk of ACS. In the comparison of CVA, patients treated with nilotinib tended to have a higher risk of CVA than those treated with imatinib. In studies where patients took more than 600 mg daily dosage of nilotinib or more than 400 mg daily dosage of imatinib, the duration of imatinib or total follow-up time was more than 5 years (Supplementary Figures 1–6).

### Results of bioinformatics analysis

#### Results of differentially expressed genes

**FIGURE 4:**
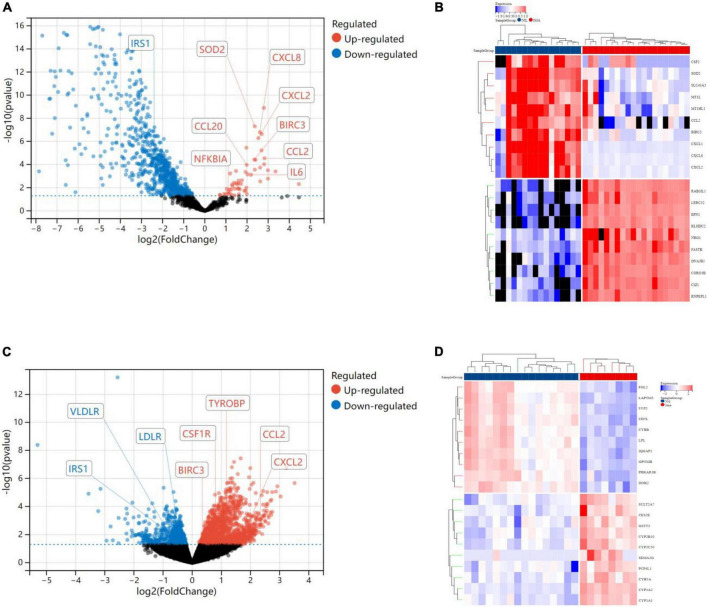
Visualized plots for differentially expressed genes (DEGs). **(A)** Volcano plots (human cardiomyocytes samples). **(B)** Heatmap (human cardiomyocytes samples). **(C)** Heatmap (liver samples from Mus musculus). **(D)** Volcanoplots (liver samples from Mus musculus).

In terms of human cardiomyocytes treated with nilotinib, 55 upregulated and 759 downregulated DEGs were identified through fold change (FC) and P value filtering (| log2FC| > 1 and *P* < 0.05) (see Figure 4). Interleukin 6 (IL6), C-X-C motif chemokine ligand 8 (CXCL8), C-C motif chemokine ligand 2 (CCL2), superoxide dismutase 2 (SOD2), NFKB inhibitor alpha (NFKBIA), baculoviral IAP repeat containing 3 (BIRC3), C-C motif chemokine ligand 20 (CCL20), and C-X-C motif chemokine ligand 2 (CXCL2) were upregulated in the nilotinib group, while insulin receptor substrate 1 (IRS1) was downregulated.

In liver samples of Mus musculus treated with nilotinib, CCL2, CXCL2, BIRC3, Transmembrane Immune Signaling Adaptor (TYROBP), and Colony Stimulating Factor 1 Receptor (CSF1R) were upregulated, while Low Density Lipoprotein Receptor (LDLR), very Low Density Lipoprotein Receptor (VLDLR), and Insulin Receptor Substrate 1 (IRS1) were downregulated compared with those treated with imatinib. TYROBP and CSF1R are important functional regulators of macrophages, which are the main inflammatory cells in vulnerable plaques and are closely related to the occurrence, development and rupture of vulnerable plaques. Decreased expression of LDLR and VLDLR in the liver can lead to hypercholesterolemia, while decreased expression of IRS1 can lead to insulin resistance (IR).

#### Weighted gene coexpression network analysis

**FIGURE 5:**
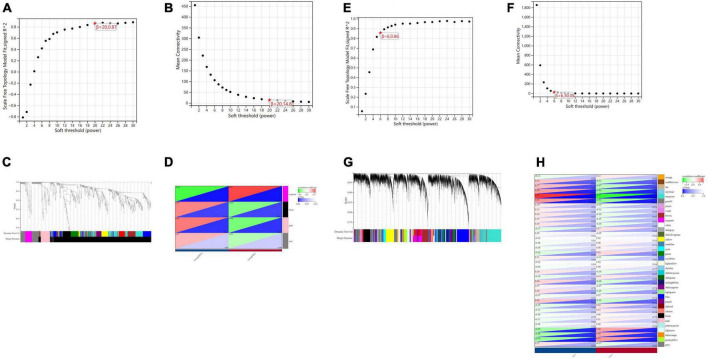
Visualization of weighted gene coexpression network analysis (WGCNA) results. **(A)** The scale-free fit index for soft-thresholding powers (human cardiomyocytes samples). **(B)** Mean connectivity (human cardiomyocytes samples). **(C)** Dendrogram of the DEGs clustered (human cardiomyocytes samples). **(D)** Heatmap showing the correlation between TKI and cardiotoxicity (human cardiomyocytes samples). **(E)** The scale-free fit index for soft-thresholding powers (liver samples from Mus musculus). **(F)** Mean connectivitys (liver samples from Mus musculus). **(G)** Dendrogram of the DEGs clustered (liver samples from Mus musculus). **(H)** Heatmap showing the correlation between TKI and atherosclerosis (liver samples from Mus musculus).

In terms of human cardiomyocyte samples, WGCNA was performed on the 814 DEGs (see Figure 5). The soft threshold for network construction was selected as 20. Meanwhile, the fitting degree of the scale-free topological model was 0.85. This network conformed to the power-law distribution and was closer to the real biological network state (41). Four modules were identified based on average linkage hierarchical clustering and soft-thresholding power. Among them, the magenta module showed the highest correlation with the cardiotoxicity of nilotinib (correlation index: 0.56, *P* = 3.4e^–4^ < 0.05). Thirty-five genes in the magenta module were selected for further analysis.

In terms of liver samples from Mus musculus, the soft threshold for network construction was selected as 6. Finally, 36 modules were identified based on average linkage hierarchical clustering and soft-thresholding power. Among them, the turquoise module showed the highest correlation with atherosclerosis related to nilotinib (correlation index: 0.77, *P* = 9.7e^–6^ < 0.05). A total of 182 genes in the turquoise module were selected for further analysis.

#### Gene ontology and kyoto encyclopedia of gene and genomes enrichment analysis

**FIGURE 6:**
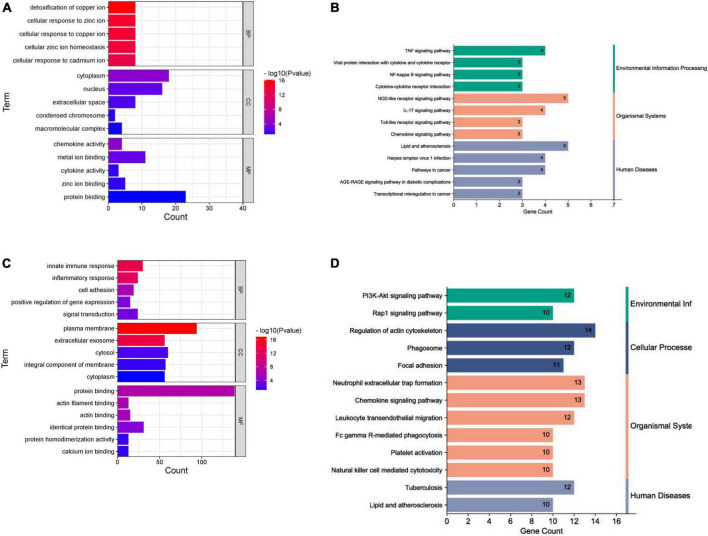
Enrichment analysis results. **(A)** GO analysis (human cardiomyocytes samples). **(B)** KEGG analysis (human cardiomyocytes samples). **(C)** GO analysis (liver samples from Mus musculus). **(D)** KEGG analysis (liver samples from Mus musculus).

In terms of human cardiomyocyte samples, 35 genes in the magenta module were analyzed by using the DAVID database. Thirty-two biological processes (BPs), 5 cellular components (CCs), and 8 molecular functions (MFs) were found. The top 5 results in terms of count with a significant difference (*P* < 0.05) and KEGG results with a count larger than 2 are presented in the bar graph according to the P value (Figures 6A,B). The smaller the P value is, the greater the color of the bar tends to be red. The greater the number of enriched genes, the longer the area of the bar was. The 35 genes were mainly associated with the NOD-like receptor signaling pathway, IL-17 signaling pathway, TNF signaling pathway, lipid and atherosclerosis, cytokine-cytokine receptor interaction and AGE-RAGE signaling pathway in diabetic complications.

In terms of liver samples from Mus musculus, genes in the turquoise module were enriched in 232 biological processes (BPs), 77-cellular components (CCs), and 8 molecular functions (MFs). In terms of KEGG analysis, genes in the turquoise module were mainly enriched in the regulation of actin cytoskeleton, chemokine signaling pathway, leukocyte transendothelial migration, PI3K-Akt, focal adhesion, Fc gamma R-mediated phagocytosis, platelet activation, natural killer cell mediated cytotoxicity, Rap1, and lipid and atherosclerosis signaling pathways (see Figures 6C,D). The results above indicated that the inflammatory response and abnormal glycolipid metabolism are the essential mechanisms in atherosclerosis related to nilotinib.

#### Protein-protein interaction network analysis

**FIGURE 7:**
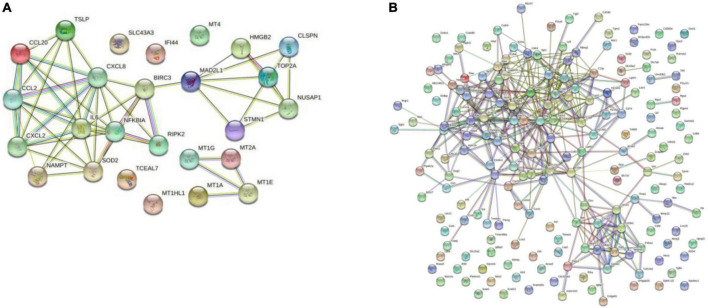
Results of the Cytoscape analysis. **(A)** PPI network (human cardiomyocytes samples). **(B)** PPI network (liver samples from Mus musculus).

The PPI network was constructed by Cytoscape based on the STRING database. In terms of human samples, the PPI network consists of 26 nodes and 54 edges (Figure 7A). The top 10 hub genes according to 5 kinds of criteria were identified by using the cytoHubba plugin (Supplementary Figure 11 and Supplementary Table 6). We took their intersection by using UpsetR (Supplementary Figure 11), and 6 hub genes were finally identified (Table 2). GO term enrichment analysis showed that the top 6 genes were enriched in the inflammatory response and signal transduction in biological processes. Cell component analysis found that they were significantly enriched in the extracellular space and extracellular region. For the molecular function analysis, they were principally involved in chemokine activity and cytokine activity. KEGG analysis suggested that they were mainly involved in the NOD-like receptor signaling pathway, IL-17, lipid and atherosclerosis pathway.

**TABLE 2:** Results of the cytoHubba analysis.

node_name	MCC	Degree	EPC	EcCentricity	Betweenness
IL6	432	10	11.80	0.27	31.47
CXCL8	432	10	11.77	0.27	31.47
CCL2	384	8	11.55	0.20	3.63
NFKBIA	288	8	11.65	0.27	14.57
SOD2	168	7	11.43	0.27	12.67
BIRC3	49	6	11.37	0.40	120.50
TYROBP	25563	27	31.03	0.19	1026.7662
CSF1R	24447	19	28.642	0.19	372.18325

In terms of samples from Mus musculus, the PPI network consists of 171 nodes and 1403 edges (Figure 7B). after taking the intersection of the top 10 hub genes according to 5 kinds of criteria, TYROBP and CSF1R were found to be hub genes, which were enriched in the osteoclast differentiation signaling pathway (Supplementary Figure 12 and Supplementary Table 7). Osteoclasts are involved in calcification formation in atherosclerotic plaques.

#### Construction of the competing endogenous RNA regulatory network for the hub genes

**FIGURE 8:**
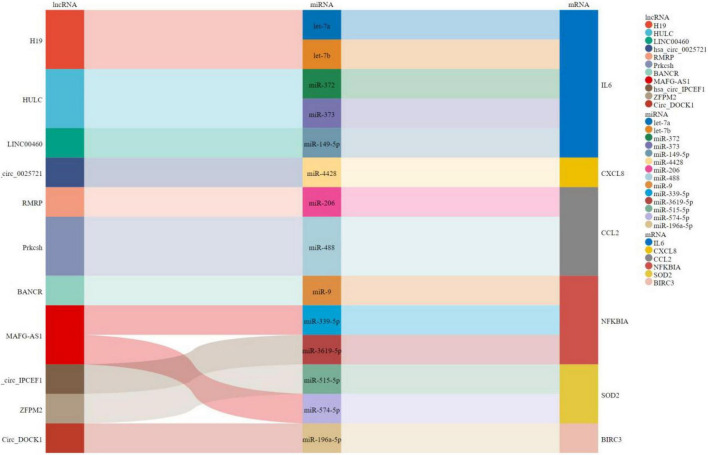
Sankey plot for ceRNAs.

As shown in Figure 8, a ceRNA coexpression network consisting of 11 lncRNAs, 14 miRNAs, and 6 mRNAs was visualized by a Sankey plot after merging these predicted results. We did not find experimentally validated ceRNAs related to TYROBP in the lncACTdb database.

#### Results of gene set enrichment analysis

Gene set enrichment analysis (GSEA) was performed to analyze the signaling pathway enrichment in the two groups. The enrichment score (ES) and normalized enrichment score (NES) were used to indicate the analysis results across gene sets. The false discovery rate (FDR) was used to judge whether a set was significantly enriched. In human cardiomyocyte samples, no pathway was found to be significantly associated with risk scores in the nilotinib group according to the criteria (| NES| > 1, FDR < 0.25, NOM *p* < 0.05).

**FIGURE 9:**
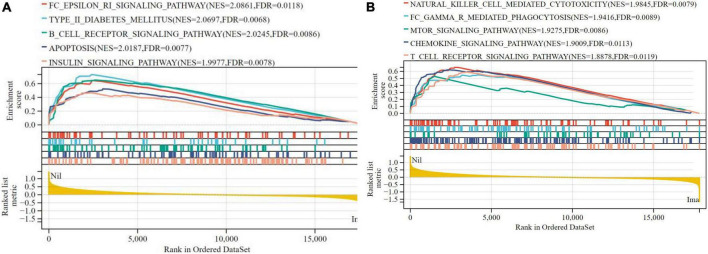
Visualized plot for the GSEA for liver samples from Mus musculus (top 10 according to NES). **(A)** Top 1–5 GSEA results and **(B)** Top 6–10 GSEA results.

In terms of liver samples from Mus musculus, 10 pathways were found to be significantly associated with risk scores in the nilotinib group, including FC epsilon RI, type II diabetes mellitus, B-cell receptor, apoptosis, insulin, natural killer cell mediated cytotoxicity, FC gamma R mediated phagocytosis, mTOR, chemokine, and T-cell receptor signaling pathways (Figure 9 and Table 3). The results indicated that nilotinib caused atherosclerosis by triggering inflammatory response and abnormal glycolipid metabolism.

**TABLE 3:** The results of GSEA.

Term	ES	NES	pvalue	FDR
FC_EPSILON_RI_SIGNALING_PATHWAY	0.6471	2.0861	0.002	0.0118
TYPE_II_DIABETES_MELLITUS	0.7333	2.0697	0.0068	0.012
B_CELL_RECEPTOR_SIGNALING_PATHWAY	0.6517	2.0245	0.0086	0.018
APOPTOSIS	0.5231	2.0187	0.0077	0.021
INSULIN_SIGNALING_PATHWAY	0.4691	1.9977	0.0078	0.025
NATURAL_KILLER_CELL_MEDIATED_CYTOTOXICITY	0.6578	1.9845	0.0079	0.03
FC_GAMMA_R_MEDIATED_PHAGOCYTOSIS	0.5508	1.9416	0.0089	0.041
MTOR_SIGNALING_PATHWAY	0.5287	1.9275	0.0021	0.0086
CHEMOKINE_SIGNALING_PATHWAY	0.6229	1.9009	0.0113	0.063
T_CELL_RECEPTOR_SIGNALING_PATHWAY	0.5899	1.8878	0.0119	0.072

#### Molecular docking simulation

**FIGURE 10:**
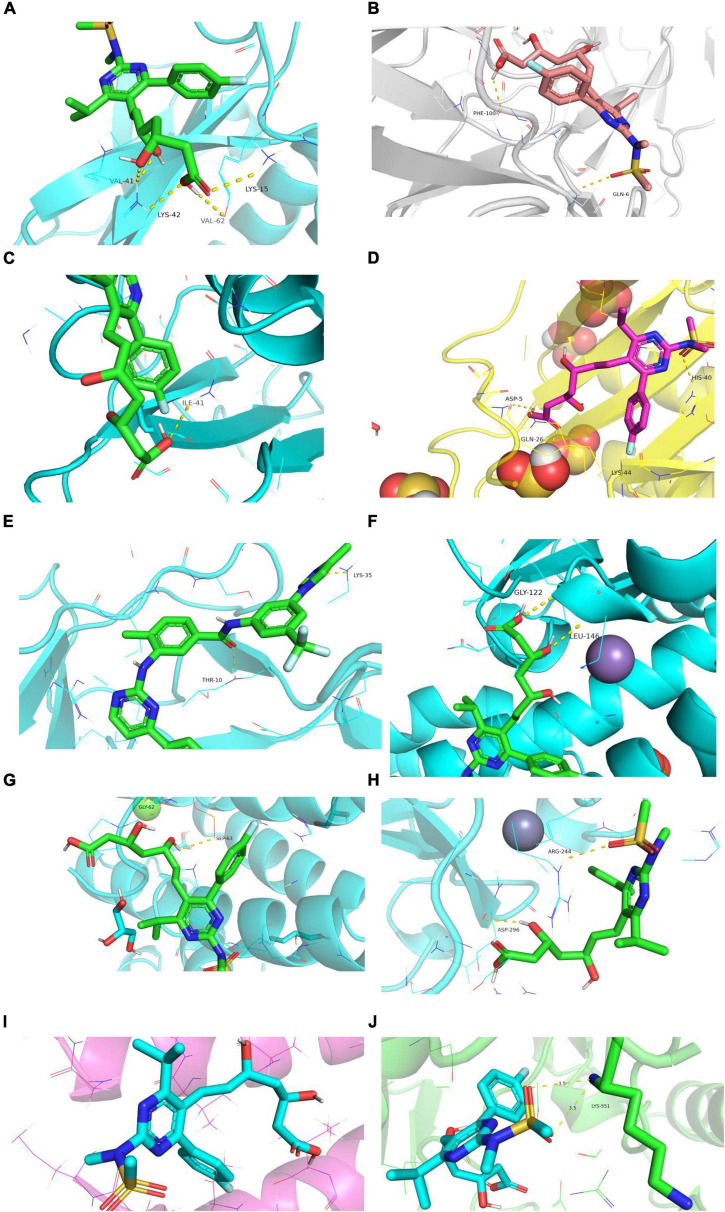
Diagram of structural interactions between rosuvastatin and hub targets. **(A)** CXCL8 **(B)** IL6 **(C)** CXCL2 **(D)** CCL20 **(E)** CCL2 **(F)** SOD2 **(G)** NFKBIA **(H)** BIRC3 **(I)** TYROBP **(J)** CSF1R.

**FIGURE 11:**
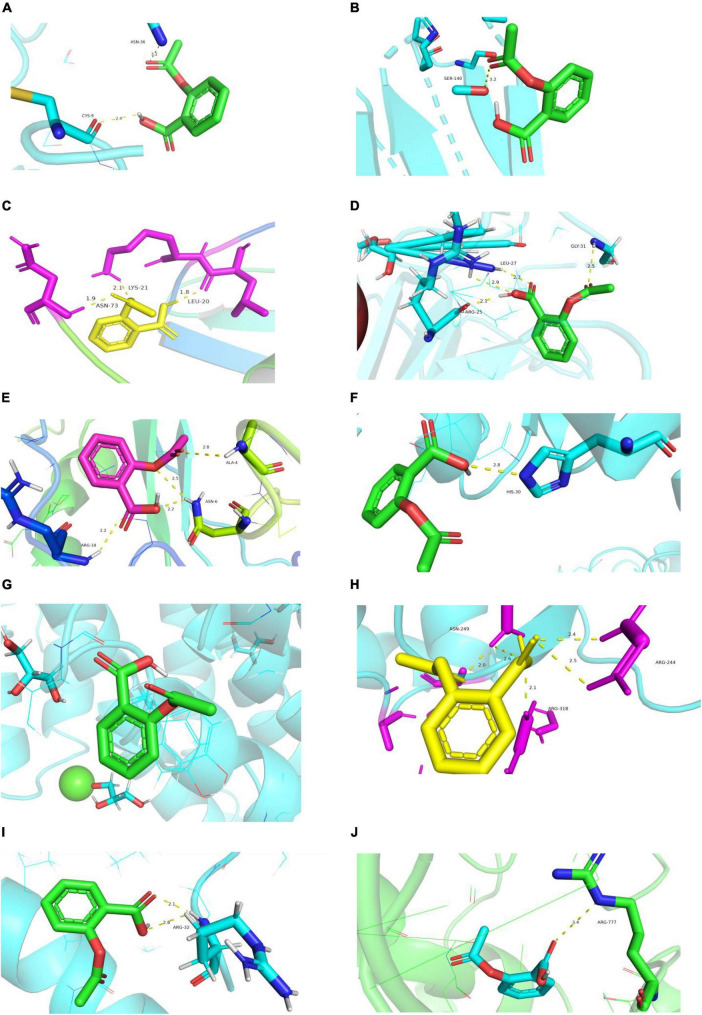
Diagram of structural interactions between aspirin and hub targets. **(A)** CXCL8 **(B)** IL6 **(C)** CXCL2 **(D)** CCL20 **(E)** CCL2 **(F)** SOD2 **(G)** NFKBIA **(H)** BIRC3 **(I)** TYROBP **(J)** CSF1R.

Rosuvastatin effectively bound to the proteins encoded by CCL20, CXCL2, NFKB1A, SOD2, BIRC3, TYROBP, and CSF1R, which were mainly enriched in the TNF and cytokine-cytokine receptor interaction signaling pathways. Aspirin could only bind to the proteins encoded by CCL20, CXCL2, and NFKB1A, which were also enriched in the TNF signaling pathway. The molecular docking scores are presented in Table 4, while the molecular docking is visualized in Figures 10, 11. The results indicated that rosuvastatin might be effective in the treatment of atherosclerosis caused by nilotinib.

**TABLE 4:** Molecular docking results in terms of the minimum binding energy (kcal/mol).

Targets	Rosuvastatin	Aspirin
CCL2	−6.7	−5.3
IL6	−4.8	−4.6
CXCL8	−4.2	−2.7
CXCL2	−5.6	−5.4
NFKB1A	−6.5	−5.8
SOD2	−5.2	−0.9
BIRC3	−5.6	−4.6
TYROBP	−5.7	−4.3
CSF1R	−6.1	−4.7

## Discussion

In this meta-analysis, we conclude that patients who start with nilotinib as first-line treatment have a higher risk of CAE, CAD, ACS, CVA, POAD and arrhythmia than those with imatinib. The evidence suggests that nilotinib is not recommended for patients with advanced age, previous cardiovascular disease or high-risk factors for CAEs. It is essential to screen for vascular risk factors, such as hypertension, hypercholesterolemia, diabetes mellitus (DM), or dyslipidemia, prior to starting nilotinib and to maintain follow-up during treatment. CML patients can be stratified according to the new Systematic Coronary Risk Evaluation (SCORE) scoring system. Patients with high and very high SCORE risk suffered from higher risk of arterial occlusive events (HR = 3.5; 95% CI = 1.4–8.7 and HR = 4.4; 95% CI = 2–9.8, respectively) (42).

Atherosclerosis has been considered the leading cause of CAD, ACS, CVA and POAD. Sudden rupture of vulnerable atherosclerotic plaques that are characterized by large necrotic cores, thin fibrous caps, calcification, and intraplaque hemorrhage can lead to acute cardiovascular adverse events (43). In this article, atherosclerosis caused by the inflammatory response and glycolipid metabolism disorder were considered the key mechanisms for the cardiotoxicity of nilotinib.

Nilotinib can upregulate the expression of cytokines and chemokines, such as CCL2, IL6, CXCL8, CXCL2, CXCL20, TYROBP and CSF1R, leading to a complex cascade that results in the formation and disruption of atherosclerotic plaques. Moreover, nilotinib downregulated the expression of LDLR, VLDLR and IRS1. LDLR is mainly involved in the catabolism of low-density lipoprotein (LDL), while VLDL is mainly involved in endogenous triglyceride transportation. Nilotinib can inhibit the ability of adipose tissue to store lipids, which results in the ectopic accumulation of fat and the development of insulin resistance (44, 45). Type 2 diabetes was also frequently observed in patients treated with nilotinib (8). As insulin receptors, downregulation of IRS1 can lead to insulin resistance, which can accelerate the decomposition of adipose tissue and increase the flow of free fatty acids (FFAs) into the liver (46), leading to the accumulation of diacylglycerol (DAG), activating protein kinase C (PKC), inhibiting the expression of IRS-1, and aggravating IR in the liver (47, 48). In the IR state, a high concentration of FFA can promote the activation of M1-type macrophages in the liver and promote the secretion of chemokines, such as CCL2 (MCP-1), TNF-α, CXCL8, CXCL2, and IL-6, which contribute to the development of atherosclerosis by regulating the activation of leukocytes, the development of foam cells and thrombosis, the proliferation of smooth muscle cells, cell egress from lesions, angiogenesis (49, 50), damage to endothelial cells and vessels (51) and the recruitment of an increasing number of monocytes and macrophages (52, 53). As a transmembrane receptor in neutrophils and monocytes/macrophages (54), TYROBP is involved in macrophage activation, lipid deposition and plaque inflammation. In the bioinformatics analysis reported by Liu et al. (55), Liu et al. (56), Zhang et al. (57), Hao and Wang (58), TYROBP was found to be one of the key Genes Involved in Advanced Atherosclerosis. CSF1R plays an important role in the survival, proliferation and differentiation of macrophages and monocytes.

However, imatinib has a positive impact on glycolipid metabolism. Imatinib can enhance the insulin-mediated vasoreactivity of resistance arteries (59), increase insulin secretion, protect against human beta-cell death (60), and reduce non-alcoholic fatty liver disease by targeting inflammatory and lipogenic pathways. Noa Markovits reported a retrospective cohort study in which long-term use of imatinib significantly reduced HbA1c (0.53%, IQR[0.09,1.19]) and FPG (10.2 mg/dL, IQR[−3.5,32.2]) in patients with diabetes, independent of demographics and glucose-lowering drug utilization, which suggested durable metabolic benefits of imatinib (61).

As the mainstream lipid-lowering drugs, statins can block cholesterol biosynthesis in liver cells enhance the intake and clearance of LDL cholesterol (LDL-C) in blood. Moreover, statins confer cardiovascular benefits through anti-inflammatory effects (62). Rosuvastatin treatment can reduce hs-CRP and IL-6 levels in patients with coronary artery ectasia (63) and inhibit the TLR4/MyD88/NF-_**K**_ B signaling pathway (64). In this article, rosuvastatin was found to bind to most of the hub genes and genes enriched in the lipid and atherosclerosis signaling pathways, which indicates that rosuvastatin may be effective in the treatment of CAE caused by nilotinib.

Our study had several limitations. First, the dosage was an important factor when discussing adverse drug reactions. Six studies did not report the dosage of nilotinib or imatinib, which might lead to some degree of heterogeneity. Second, some studies did not introduce the risk factors or previous history of cardiovascular events of patients included, which might lead to some degree of bias. Third, vascular endothelial cells or cardiomyocytes from CML patients treated with nilotinib or imatinib may provide more information about atherosclerosis related to nilotinib, but no dataset in this respect was found in the GEO database. Fourth, bioinformatics analysis and molecular docking can only suggest the potential mechanism and potential therapeutic drugs, which lacks experimental validation. We will conduct relevant experiments in the future.

## Conclusion

This meta-analysis suggests that patients who start with nilotinib as first-line treatment have a higher risk of cardiovascular adverse events than those with imatinib. Atherosclerosis caused by the inflammatory response and glycolipid metabolism disorders are the key mechanisms of nilotinib cardiotoxicity. Rosuvastatin may be beneficial in the treatment of CAE caused by nilitinib.

## Author contributions

XN, LS, and JL conceived of the study and design. XN conceived the early ideas for the application of the analysis models and worked on the critical revision of the manuscript. SL and JH collected data, led the analysis and interpretation of findings, and drafted the initial and subsequent versions of the manuscript. XZ and YC helped with data extraction and interpretation. All authors contributed to the analysis and interpretation of the data, revised the manuscript for important intellectual content, and contributed to the manuscript as presented here.

## Footnotes

1
https://www.ncbi.nlm.nih.gov/gds/

2
https://david.ncifcrf.gov/

3
http://www.bioinformatics.com.cn/

4
https://www.string-db.org

5
http://www.biobigdata.ta.net/LncACTdb/

6
http://software.broadinstitute.org/gsea/index.jsp

7
http://www.gsea-msigdb.org/gsea/downloads.jsp

8
https://pubchem.ncbi.nlm.nih.gov/

9
http://www.rcsb.org/

## Abbreviations

ACS: Acute coronary syndrome
BIRC3: Baculoviral IAP Repeat Containing 3
BP: biological process
CAE: Cardiovascular Adverse events
CCL2: C-C Motif Chemokine Ligand 2
CCL20: C-C Motif Chemokine Ligand 20
CC: cellular component
CVA: cerebrovascular accident
CNKI: China National Knowledge Internet
CML: chronic myeloid leukemia
ceRNA: competing endogenous RNA
CAD: coronary artery disease
CXCL2: C-X -C Motif Chemokine Ligand 2
CXCL8: C-X -C Motif Chemokine Ligand 8
DM: diabetes mellitus
DEGs: differentially expressed genes
ES: enrichment score
FDR: false discovery rate
FC: fold change
GEO: Gene Expression Omnibus database
GO: Gene Ontology
GSEA: Gene Set Enrichment Analysis
HF: heart failure
hs-CRP: high-sensitivity C-reactive protein
HTN: hypertension
IGM: impaired glucose metabolism
IL10: interleukin 10
IL6: Interleukin 6
IHD: ischemic heart disease
KEGG: Kyoto Encyclopedia of Gene and Genomes
ME: module eigengene
MM: module membership
MF: molecular function
NFKBIA: NFKB Inhibitor Alpha
NILO: Nilotinib
NES: normalized enrichment score
PAOD: peripheral artery occlusive disease
PRISMA: Preferred Reporting Items for Systematic Reviews and Meta-Analyses
PDB: Protein Data Bank
PPI: protein-protein interaction
PH: pulmonary hypertension
RCT: randomized controlled trial
SOD2: Superoxide Dismutase 2
TIA: transient ischemic attack
TNFα: Tumor Necrosis Factor-α
TKIs: tyrosine kinase inhibitors
VSMCs: vascular smooth muscle cells
WGCNA: weighted gene coexpression network analysis.
