# Supplementary material for: Cardiovascular adverse events in chronic myeloid leukemia patients treated with nilotinib or imatinib: A systematic review, meta-analysis and integrative bioinformatics analysis
Source: Front Cardiovasc Med. 2022 Nov 8;9:966182. doi: 10.3389/fcvm.2022.966182 (PMC9678945; doi:10.3389/fcvm.2022.966182)
Supplement: Supplementary file 1 [file Data_Sheet_1.docx]

**Supplementary materials**

Table 1 English literature search strategy(Pubmed)

| **Number** | **Search terms** |
| --- | --- |
| 1 | imatinib [Title/Abstract] |
| 2 | imatinib[MeSH Terms] |
| 3 | 1 OR 2 |
| 4 | Nilotinib [Title/Abstract] |
| 5 | Nilotinib[MeSH Terms] |
| 6 | 4 OR 5 |
| 7 | 3 AND 6 |
| 8 | Chronic myeloid leukemia[Title/Abstract] |
| 9 | Chronic myeloid leukemia[MeSH Terms] |
| 10 | Chronic Myelogenous Leukemia[Title/Abstract] |
| 11 | Chronic Myelogenous Leukemia[MeSH Terms] |
| 12 | Chronic Myelocytic Leukemia[Title/Abstract] |
| 13 | Chronic Myelocytic Leukemia[MeSH Terms] |
| 14 | Chronic Granulocytic Leukemia[Title/Abstract] |
| 15 | Chronic Granulocytic Leukemia[MeSH Terms] |
| 16 | Philadelphia-Positive Myeloid Leukemia[Title/Abstract] |
| 17 | Philadelphia-Positive Myeloid Leukemia[MeSH Terms] |
| 18 | Ph1-Positive Myelogenous Leukemia[Title/Abstract] |
| 19 | Ph1-Positive Myelogenous Leukemia[MeSH Terms] |
| 20 | Ph1-Positive Myeloid Leukemia[Title/Abstract] |
| 21 | Ph1-Positive Myeloid Leukemia[MeSH Terms] |
| 22 | 8 OR 9 OR 10 OR 11 OR 12 OR 13 OR 14 OR 15 OR 16 OR 17 OR 18 OR 19 OR 20 OR 21 |
| 23 | Cardio* [Title/Abstract] |
| 24 | Vascular [Title/Abstract] |
| 25 | Arterial [Title/Abstract] |
| 26 | Myocardial [Title/Abstract] |
| 27 | Atherosclerosis [Title/Abstract] |
| 28 | Coronary [Title/Abstract] |
| 29 | Angina [Title/Abstract] |
| 30 | Cardiac [Title/Abstract] |
| 31 | Cardiotoxicity [MeSH Terms] |
| 32 | Heart [Title/Abstract] |
| 33 | Occlusive [Title/Abstract] |
| 34 | Ischemic [Title/Abstract] |
| 35 | 23 OR 24 OR 25 OR 26 OR 27 OR 28 OR 29 OR 30 OR 31 OR 32 OR 33 OR 34 |
| 36 | 7 AND 22 AND 35 |

Table 2 English literature search strategy(Embase)

| **Number** | **Search terms** |
| --- | --- |
| 1 | imatinib [Title/Abstract] |
| 2 | Nilotinib [Title/Abstract] |
| 3 | 1 OR 2 |
| 4 | Chronic myeloid leukemia[Title/Abstract] |
| 5 | Chronic Myelogenous Leukemia[Title/Abstract] |
| 6 | Chronic Myelocytic Leukemia[Title/Abstract] |
| 7 | Chronic Granulocytic Leukemia[Title/Abstract] |
| 8 | Philadelphia-Positive Myeloid Leukemia[Title/Abstract] |
| 9 | Ph1-Positive Myelogenous Leukemia[Title/Abstract] |
| 10 | Ph1-Positive Myeloid Leukemia[Title/Abstract] |
| 11 | 4 OR 5 OR 6 OR 7 OR 8 OR 9 OR 10 |
| 12 | Ischemic [Title/Abstract] |
| 13 | Cardio* [Title/Abstract] |
| 14 | Vascular [Title/Abstract] |
| 15 | Arterial [Title/Abstract] |
| 16 | Myocardial [Title/Abstract] |
| 17 | Atherosclerosis [Title/Abstract] |
| 18 | Coronary [Title/Abstract] |
| 19 | Angina [Title/Abstract] |
| 20 | Cardiac [Title/Abstract] |
| 21 | Cardiotoxicity [MeSH Terms] |
| 22 | Heart [Title/Abstract] |
| 23 | Occlusive [Title/Abstract] |
| 24 | 12 OR 13 OR 14 OR 15 OR 16 OR 17 OR 18 OR 19 OR 20 OR 21 OR 22 OR 23 |
| 25 | 3 AND 11 AND 24 |

Table3 English literature search strategy( Cochrane library)

| **Number** | **Search terms** |
| --- | --- |
| 1 | imatinib [Title Abstract Keyword] |
| 2 | Nilotinib [Title Abstract Keyword] |
| 3 | 1 OR 2 |
| 4 | Chronic myeloid leukemia[Title Abstract Keyword] |
| 5 | Chronic Myelogenous Leukemia[Title Abstract Keyword] |
| 6 | Chronic Myelocytic Leukemia[Title Abstract Keyword] |
| 7 | Chronic Granulocytic Leukemia[Title Abstract Keyword] |
| 8 | Philadelphia-Positive Myeloid Leukemia[Title Abstract Keyword] |
| 9 | Ph1-Positive Myelogenous Leukemia[Title Abstract Keyword] |
| 10 | Ph1-Positive Myeloid Leukemia[Title Abstract Keyword] |
| 11 | 4 OR 5 OR 6 OR 7 OR 8 OR 9 OR 10 |
| 12 | Ischemic [Title Abstract Keyword] |
| 13 | Cardio* [Title Abstract Keyword] |
| 14 | Vascular [Title Abstract Keyword] |
| 15 | Arterial [Title Abstract Keyword] |
| 16 | Myocardial [Title Abstract Keyword] |
| 17 | Atherosclerosis [Title Abstract Keyword] |
| 18 | Coronary [Title Abstract Keyword] |
| 19 | Angina [Title Abstract Keyword] |
| 20 | Cardiac [Title Abstract Keyword] |
| 21 | Cardiotoxicity [MeSH Terms] |
| 22 | Heart [Title Abstract Keyword] |
| 23 | Occlusive [Title Abstract Keyword] |
| 24 | 12 OR 13 OR 14 OR 15 OR 16 OR 17 OR 18 OR 19 OR 20 OR 21 OR 22 OR 23 |
| 25 | 3 AND 11 AND 24 |

As was showed in the Table 4, P-value of Begg’s and Egger’s test was all above 0.05, so no significant publication bias was found, expect for the two outcomes(HF and arrhythmia ).

Table 4 Publication bias assessment

|  | Number of studies | P-value of Begg’s test | P-value of Egger’s test |
| --- | --- | --- | --- |
| CAE-OR | 14 | 0.2983 | 0.2875 |
| CAE-HR | 2 | -- | -- |
| CAD | 9 | 0.6767 | 0.7176 |
| ACS | 6 | 0.3476 | 0.7479 |
| CVA | 9 | 0.1376 | 0.0494 |
| PAOD | 10 | 0.8153 | 0.1037 |
| HF | 3 | -- | -- |
| Arrhythmia | 2 | -- | -- |

**Table5 Quality assessment of included studies according to the Newcastle-Ottawa Scale.**

| **Study** | **Selection** | | | | **Comparability** | | **Outcome** | | | **In total** |
| --- | --- | --- | --- | --- | --- | --- | --- | --- | --- | --- |
|  | 1^*^ | 2^*^ | 3^*^ | 4^*^ | 5^*^ | 6^*^ | 7^*^ | 8^*^ | 9^*^ |  |
| Isao Fujioka 2018 | 1 | 1 | 1 | 1 | 1 | 0 | 1 | 1 | 1 | 8 |
| Torsten Dahlén 2022 | 1 | 1 | 1 | 1 | 1 | 0 | 1 | 1 | 1 | 8 |
| Petrikova L 2021 | 1 | 1 | 1 | 1 | 1 | 0 | 1 | 1 | 0 | 7 |
| Torsten Dahlen 2016 | 1 | 1 | 1 | 1 | 1 | 0 | 1 | 0 | 1 | 7 |
| Mei-Tsen Chen 2021 | 1 | 1 | 1 | 1 | 1 | 0 | 1 | 1 | 1 | 8 |
| Preetesh Jain  2021 | 1 | 1 | 1 | 1 | 1 | 0 | 1 | 1 | 1 | 8 |
| Masahiro Kizaki 2019 | 1 | 1 | 1 | 1 | 1 | 0 | 1 | 1 | 1 | 8 |
| F J Giles 2013 | 1 | 1 | 1 | 1 | 1 | 0 | 0 | 1 | 1 | 7 |
| Yu-Chien Yang 2021 | 1 | 1 | 1 | 1 | 1 | 0 | 1 | 0 | 1 | 7 |
| Kantarjian HM 2021 | 1 | 1 | 1 | 1 | 1 | 0 | 1 | 1 | 1 | 8 |
| Anna Sicuranza 2022 | 1 | 1 | 1 | 1 | 1 | 0 | 1 | 0 | 1 | 7 |
| T D Kim 2013 | 1 | 1 | 1 | 1 | 1 | 0 | 1 | 1 | 1 | 8 |
| Jianxiang Wang 2015 | 1 | 1 | 1 | 1 | 1 | 0 | 1 | 0 | 1 | 7 |
| Alessandra Bettiol 2018 | 1 | 1 | 1 | 1 | 1 | 0 | 1 | 1 | 0 | 7 |

1^*^: Representativeness of the exposed cohort, 2^*^: Selection of the non exposed cohort, 3^*^: Ascertainment of exposure, 4^*^: Demonstration that outcome of interest was not present at start of study, 5^*^: Study controls for the most important factor, 6^*^: Study controls for any additional factor, 7^*^: Assessment of outcome, 8^*^: Was follow-up long enough for outcomes to occur, 9^*^: Adequacy of follow up of cohorts.

Table 6 TOP 10 hub genes according to 5 kinds of criteria( human cardiomyocytes)

| Rank | MCC | Degree | EPC | Betweenness | EcCentricity |
| --- | --- | --- | --- | --- | --- |
| 1 | CXCL8 | CXCL8 | IL6 | BIRC3 | BIRC3 |
| 2 | IL6 | IL6 | CXCL8 | MAD2L1 | MAD2L1 |
| 3 | CCL2 | CCL2 | NFKBIA | IL6 | IL6 |
| 4 | CXCL2 | NFKBIA | CCL2 | CXCL8 | CXCL8 |
| 5 | NFKBIA | CXCL2 | CXCL2 | NFKBIA | NFKBIA |
| 6 | CCL20 | SOD2 | SOD2 | SOD2 | SOD2 |
| 7 | SOD2 | CCL20 | BIRC3 | MT1E | RIPK2 |
| 8 | TSLP | BIRC3 | CCL20 | CCL2 | CCL2 |
| 9 | BIRC3 | MAD2L1 | TSLP | NUSAP1 | NUSAP1 |
| 10 | NAMPT | TSLP | RIPK2 | TOP2A | TOP2A |

Table 7 TOP 10 hub genes according to 5 kinds of criteria(Mus musculus liver samples)

| Rank | MCC | Degree | EPC | Betweenness | EcCentricity |
| --- | --- | --- | --- | --- | --- |
| 1 | Tyrobp | Ptprc | Ptprc | Cd44 | Ptprc |
| 2 | Csf1r | Itgb2 | Itgb2 | Ptprc | Itgax |
| 3 | Ctss | Tyrobp | Tyrobp | Actb | Cd44 |
| 4 | C1qa | Csf1r | Csf1r | Itgb2 | Tlr2 |
| 5 | C1qb | Ctss | Ctss | C1qa | Icam1 |
| 6 | C1qc | Cd44 | C1qa | Tyrobp | Lgals3 |
| 7 | Cd68 | Rac2 | Cd68 | Tlr2 | Bgn |
| 8 | Aif1 | Actb | C1qb | Dcn | Itgb2 |
| 9 | Ly86 | C1qa | Itgax | Pik3cd | Tyrobp |
| 10 | Lyz2 | C1qb | Vav1 | Col1a1 | Csf1r |

| A  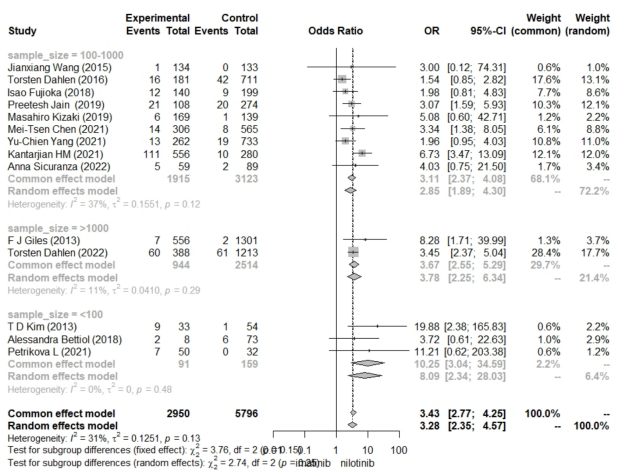 | B  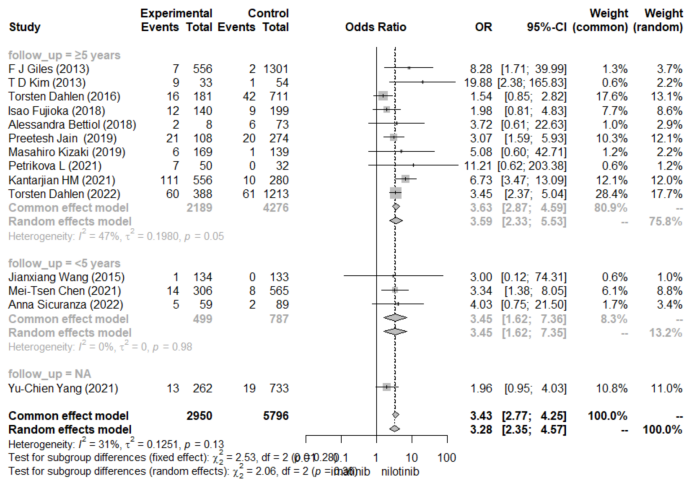 |
| --- | --- |
| C  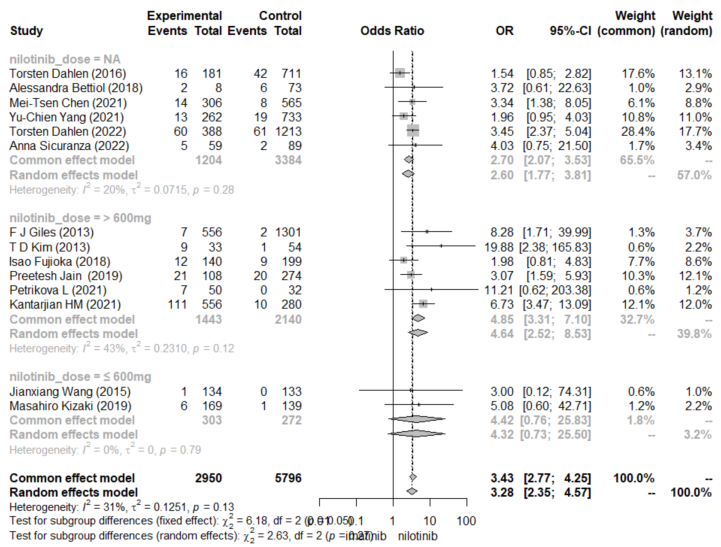 | D  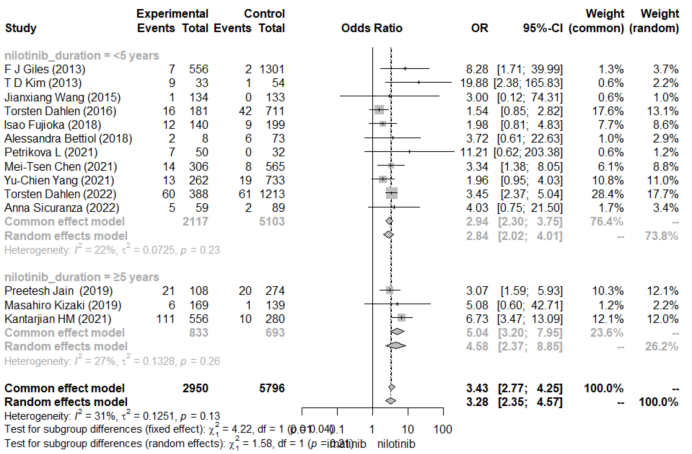 |
| E.  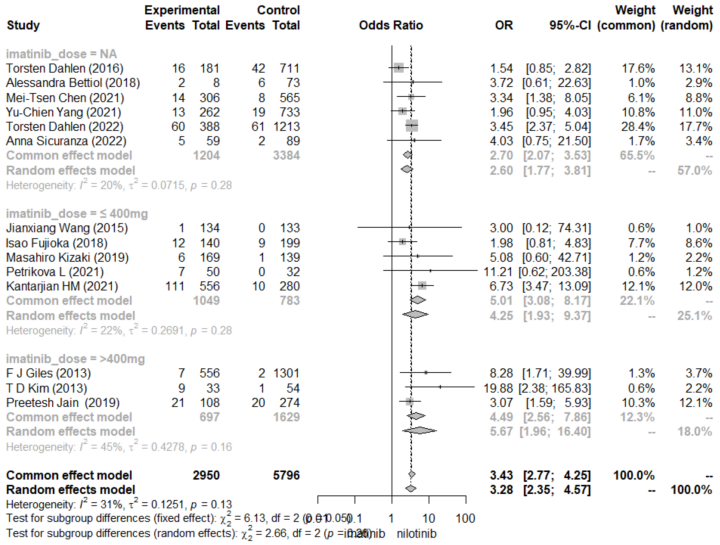 | F.  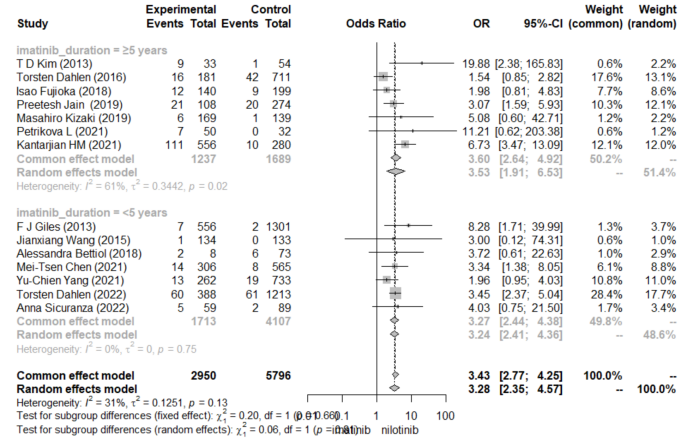 |
| G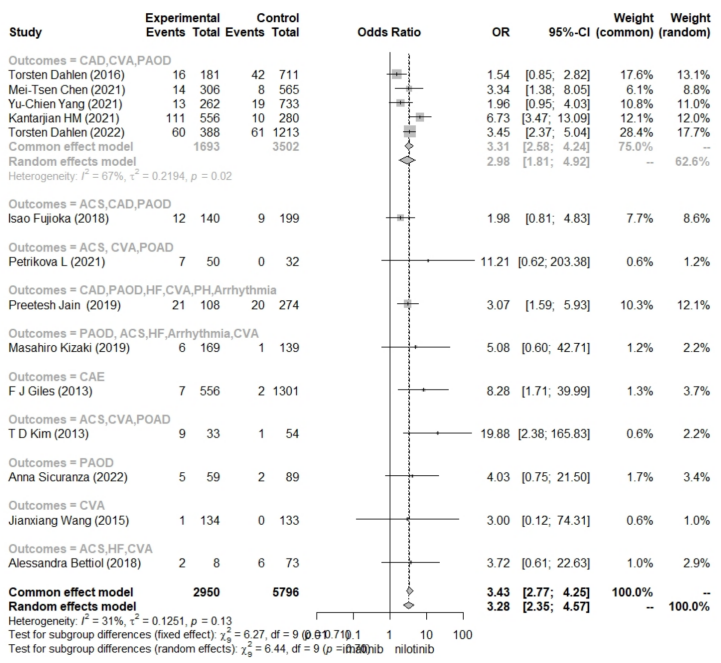 |  |

Figure 1 Subgroup analysis for CAE (A.sample size B. median follow-up time C.nilotinib dose D. nilotinib duration E. imatinib_dose F. imatinib_duration G. outcomes)

| A  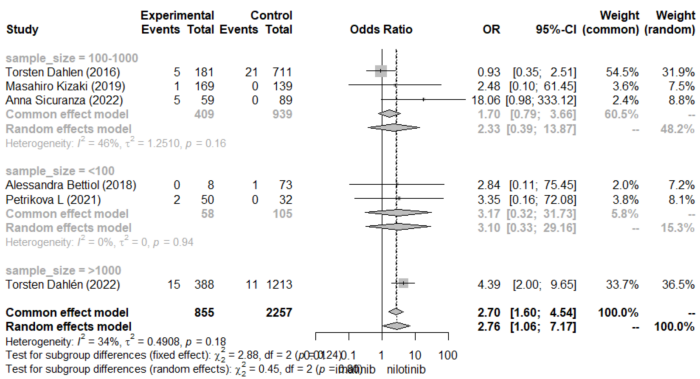 | B  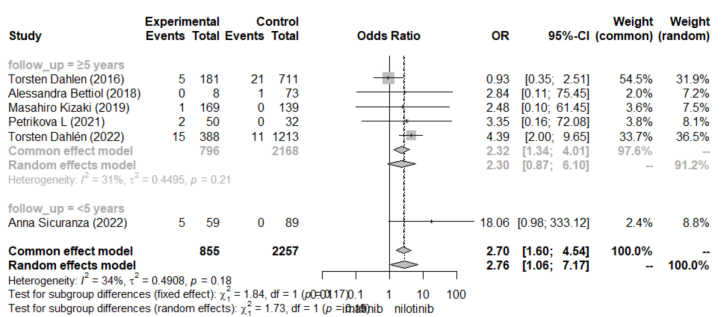 |
| --- | --- |
| C  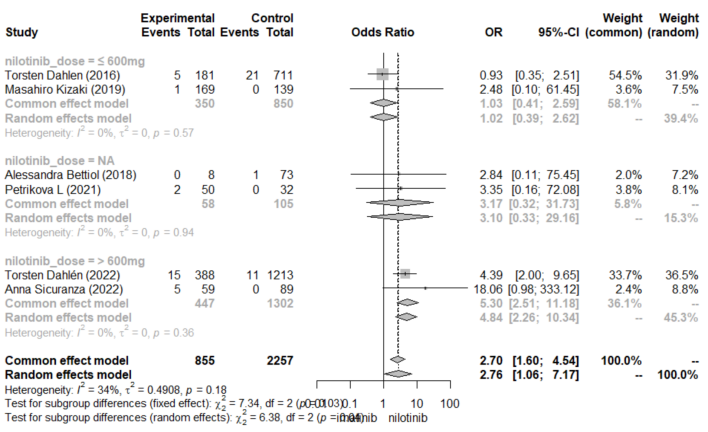 | D  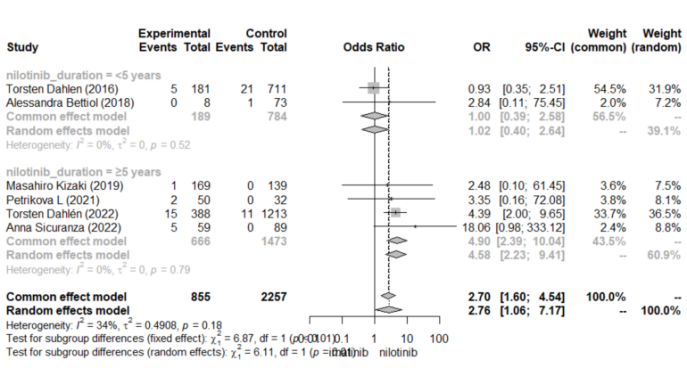 |
| E  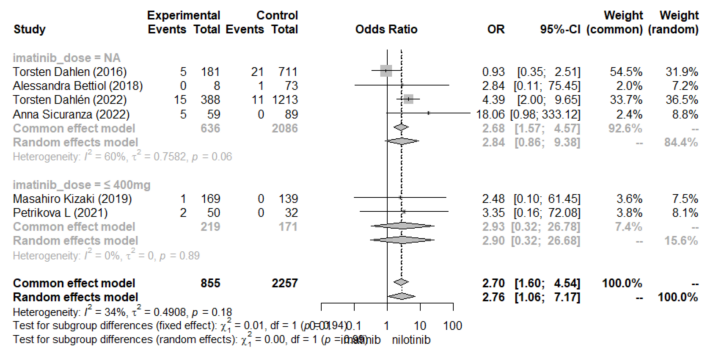 | F  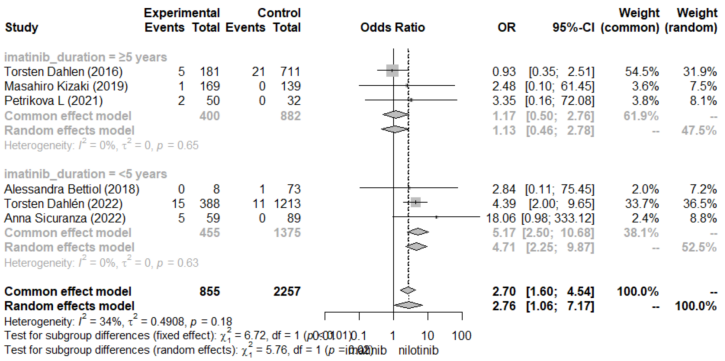 |

Figure 2 Subgroup analysis for ACS (A.sample size B. median follow-up time C.nilotinib dose D. nilotinib duration E. Imatinib_dose F. Imatinib_duration )

| A  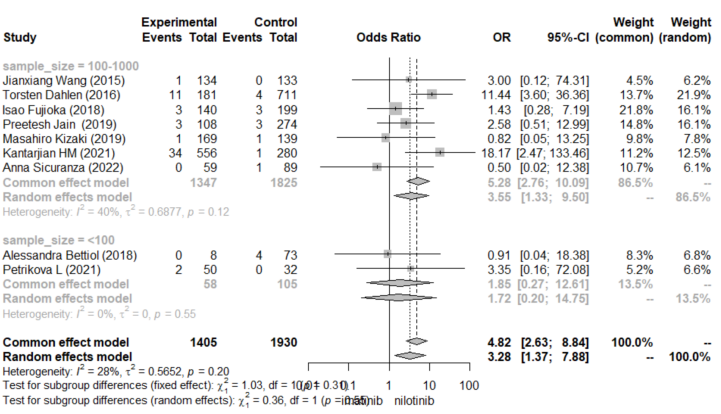 | B  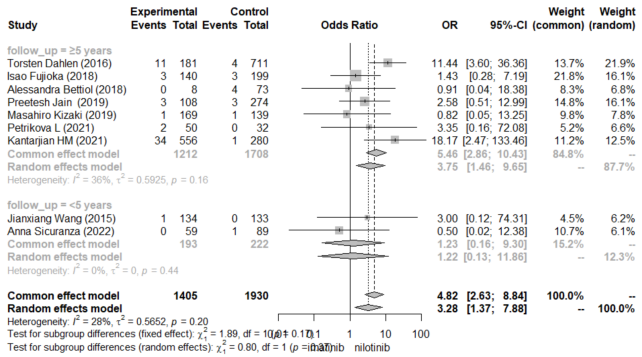 |
| --- | --- |
| C  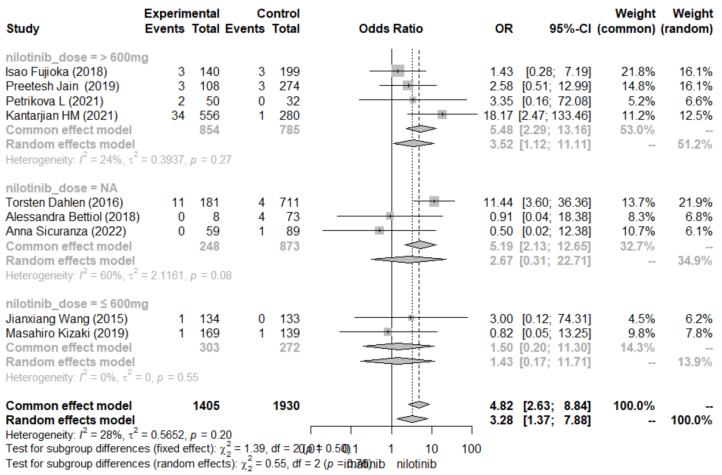 | D  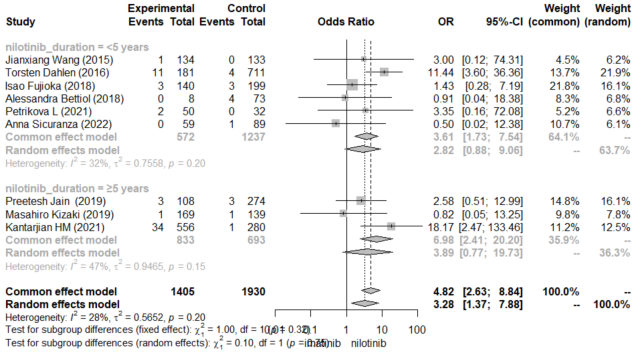 |
| E  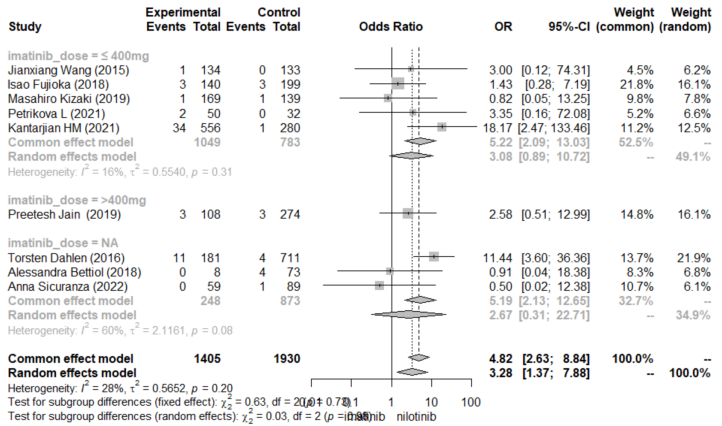 | F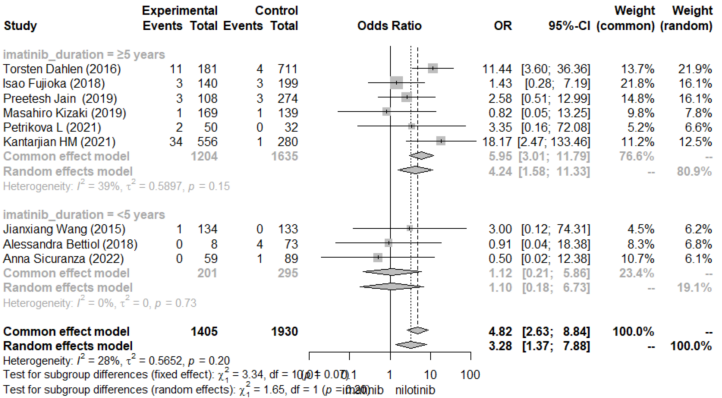 |

Figure 3 Subgroup analysis for CVA (A.sample size B. median follow-up time C.nilotinib dose D. nilotinib duration E. Imatinib_dose F. Imatinib_duration)

| A  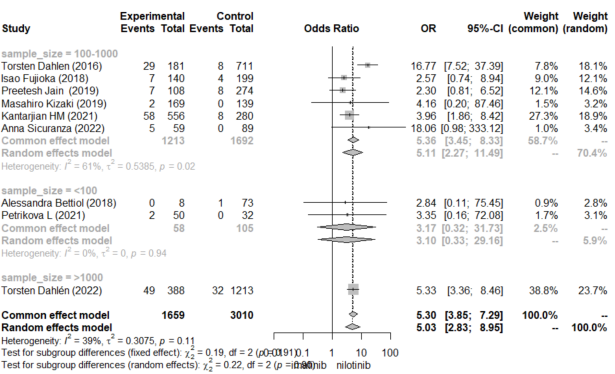 | B  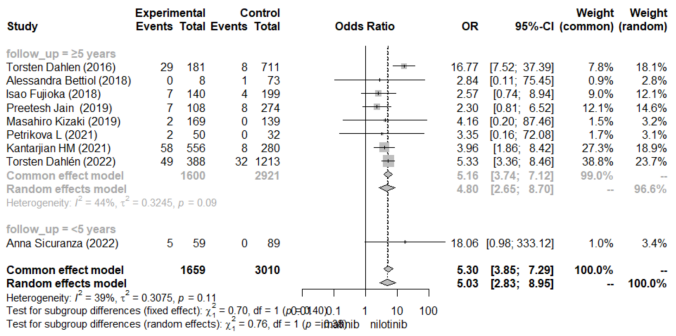 |
| --- | --- |
| C  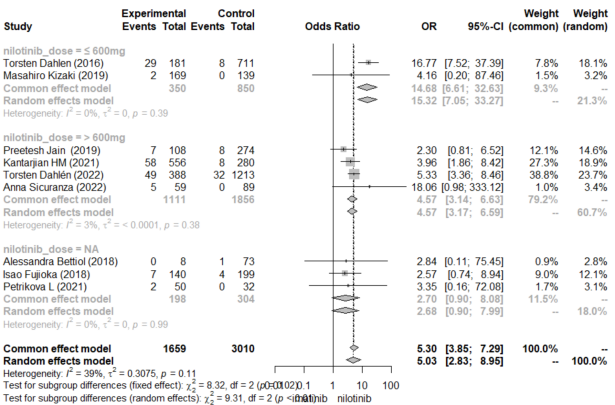 | D  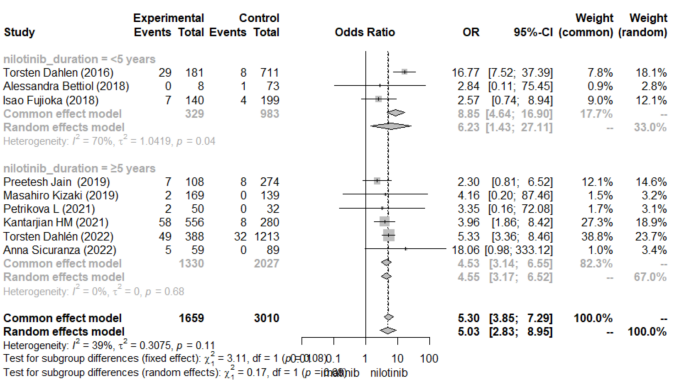 |
| E  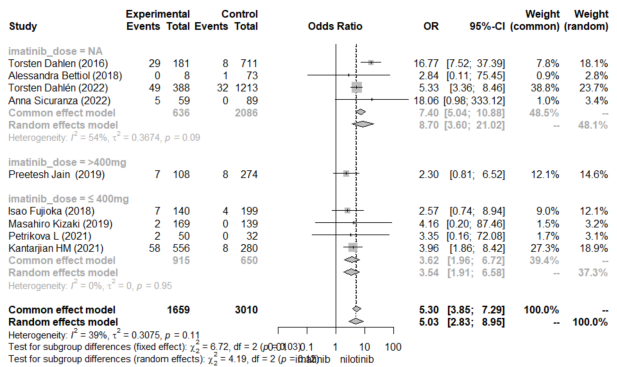 | F  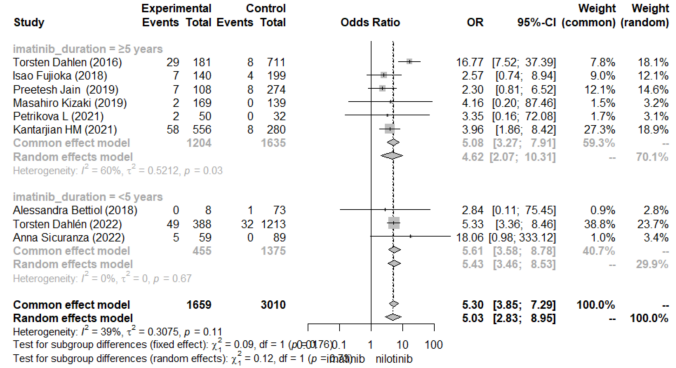 |

Figure 4 Subgroup analysis for CAD (A.sample size B. median follow-up time C.nilotinib dose D. nilotinib duration E. Imatinib_dose F. Imatinib_duration )

| A  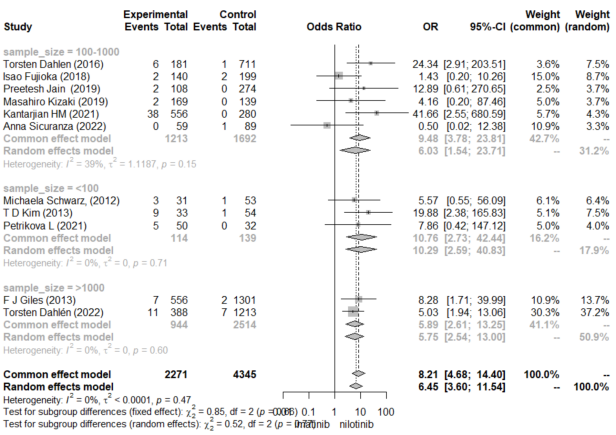 | B  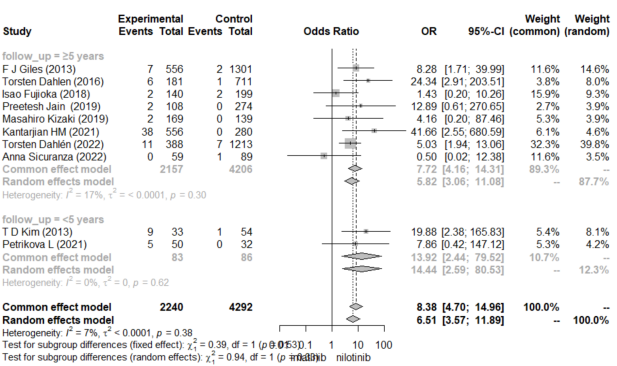 |
| --- | --- |
| C  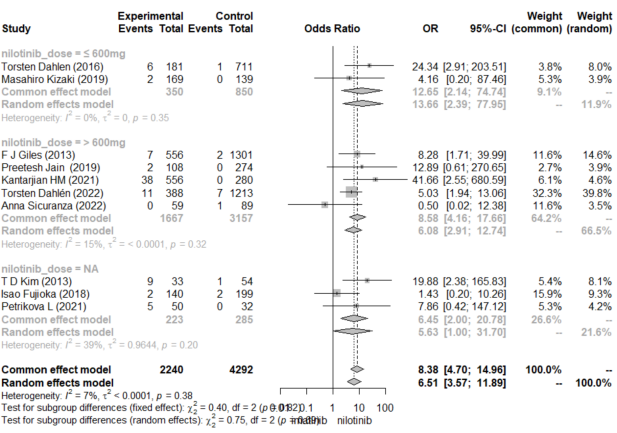 | D  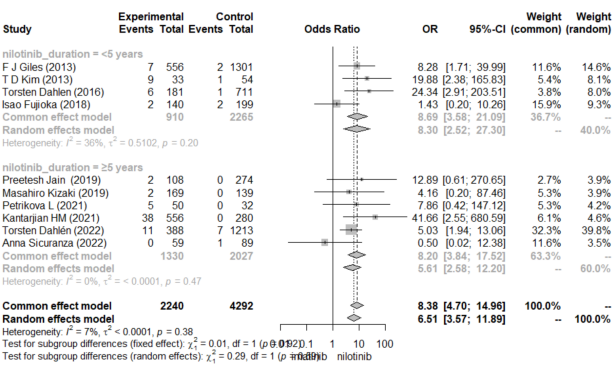 |
| E  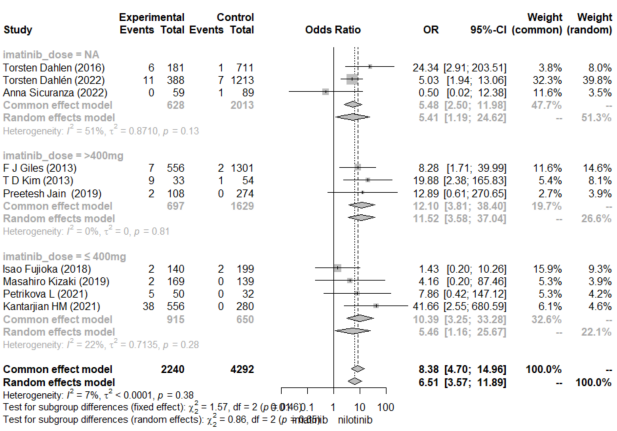 | F  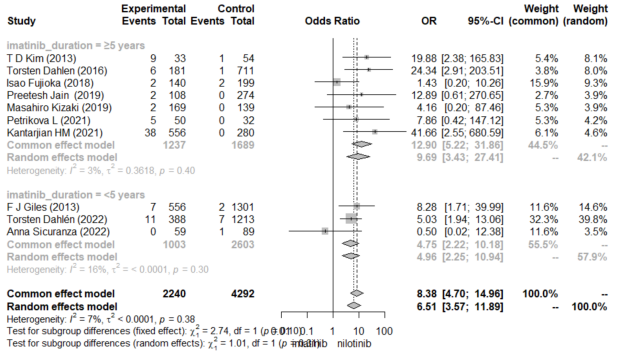 |

Figure 5 Subgroup analysis for PAOD (A.sample size B. median follow-up time C.nilotinib dose D. nilotinib duration E. Imatinib_dose F. Imatinib_duration )

| A  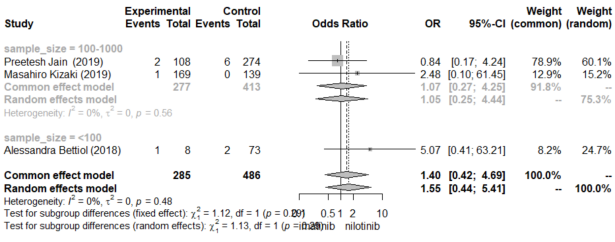 | B  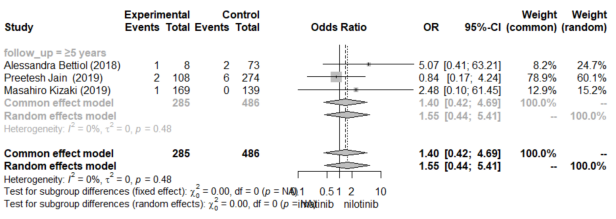 |
| --- | --- |
| C  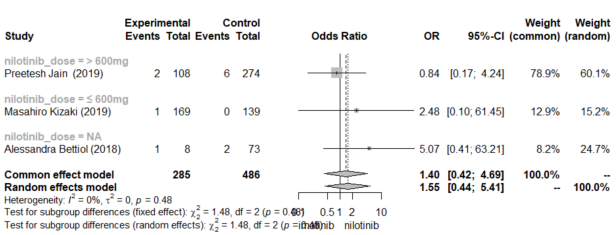 | D  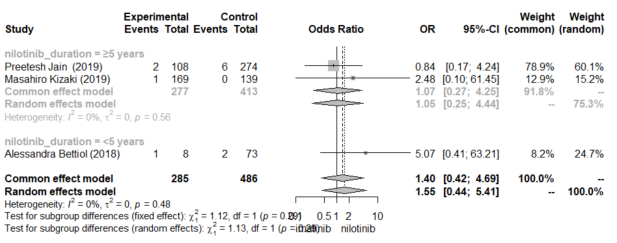 |
| E  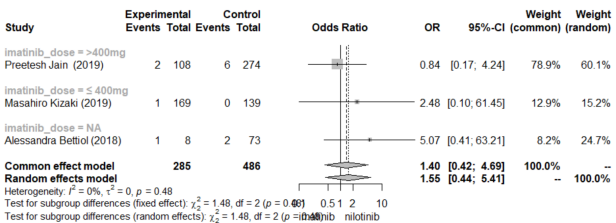 | F  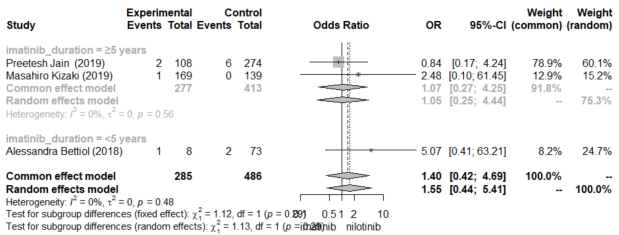 |

Figure 6 Subgroup analysis for HF (A.sample size B. median follow-up time C.nilotinib dose D. nilotinib duration E. imatinib_dose F. imatinib_duration)

| A.  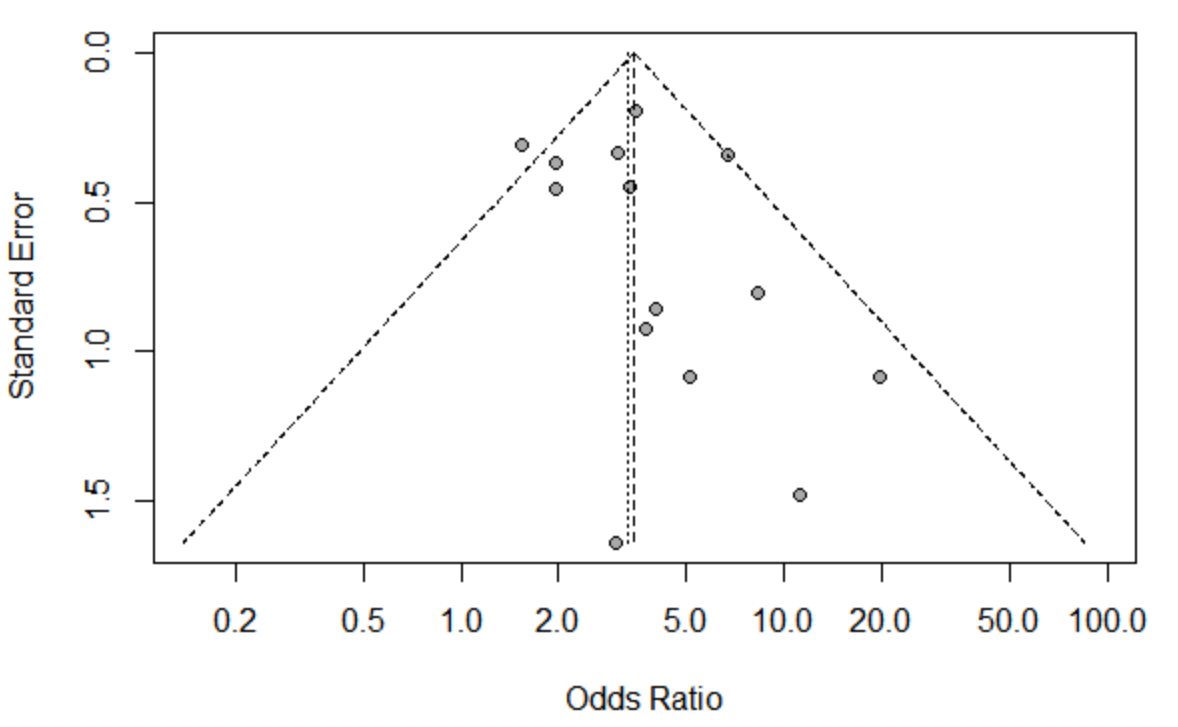 | B.  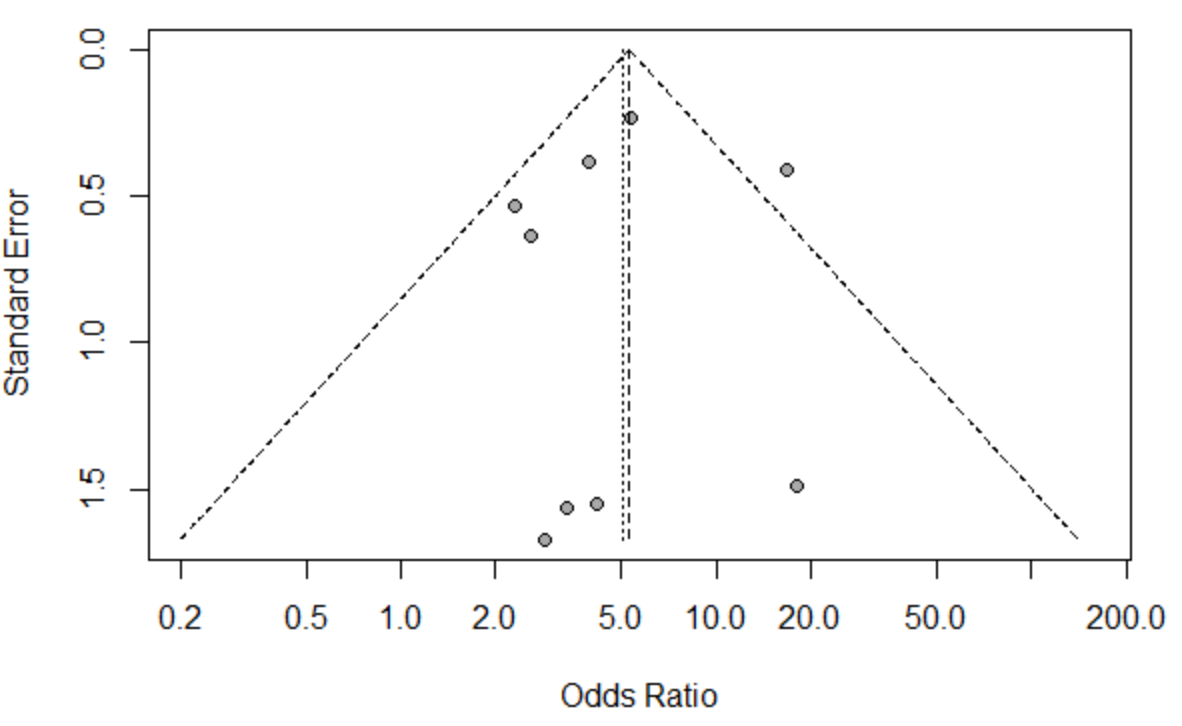 |
| --- | --- |
| C.  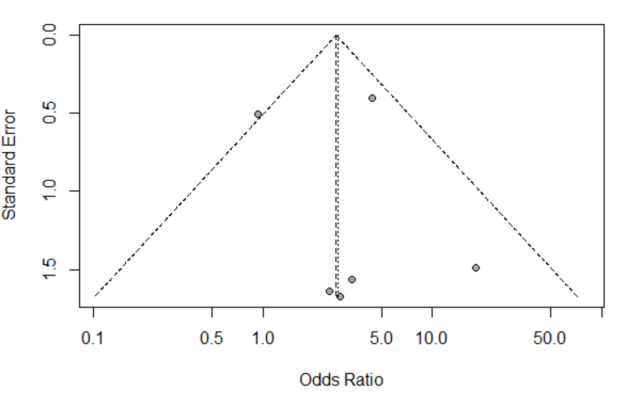 | D.  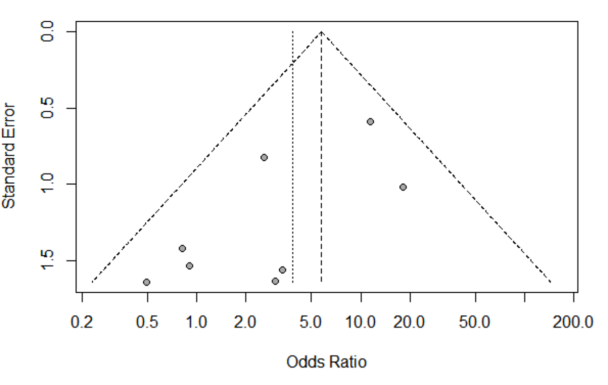 |
| E.  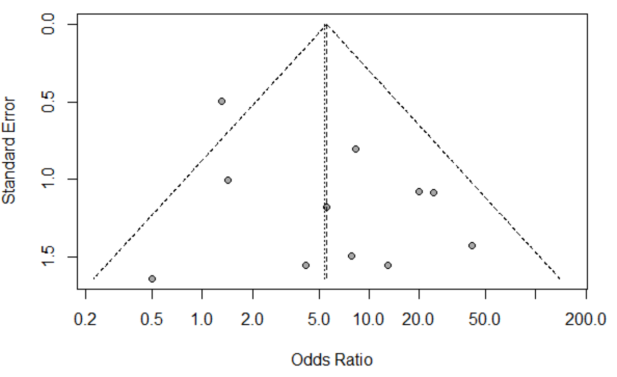 | 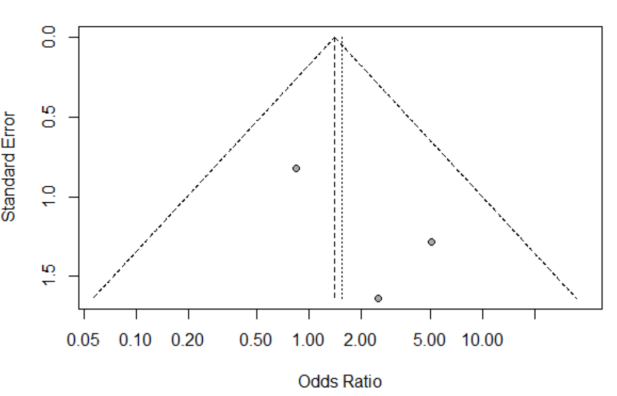 |

Figure 7 Begg’s forest plots ( A. CAE-OR B. CAE-HR C. CAD D. ACS E. CVA F.PAOD G. HF H.Arrhythmia)

| A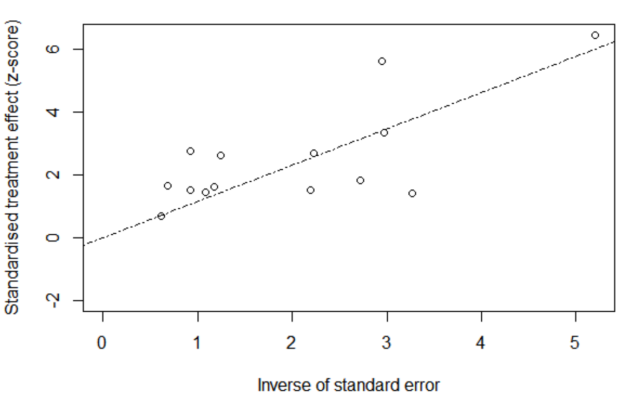 | B  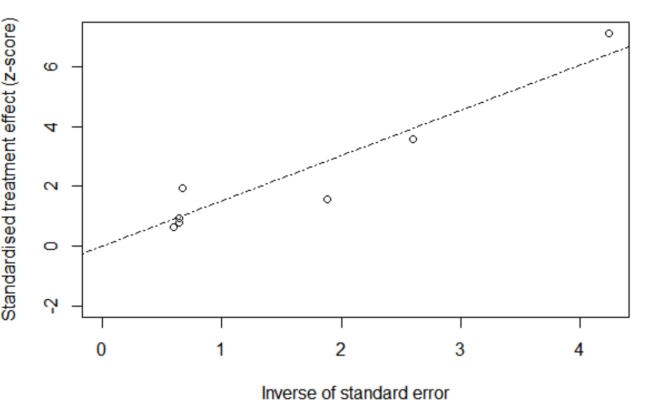 |
| --- | --- |
| C  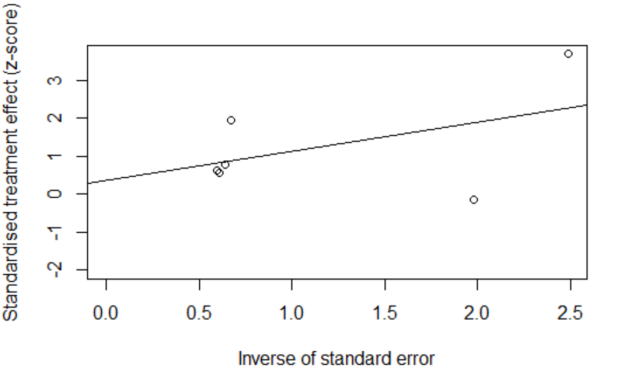 | D  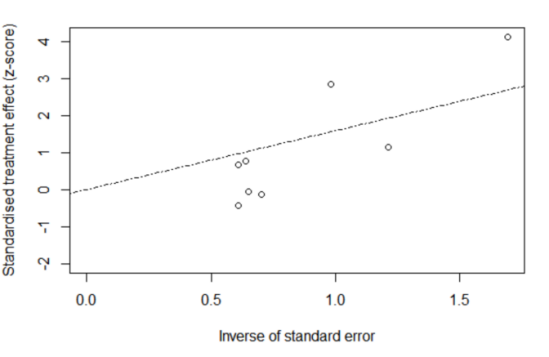 |
| E  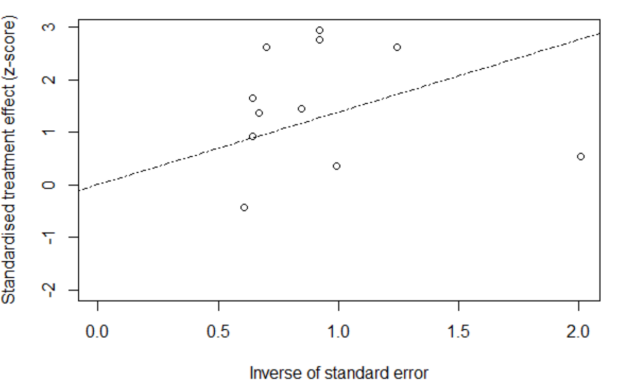 | F  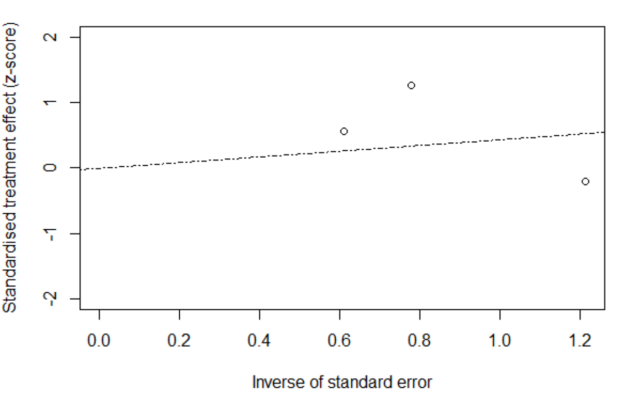 |

Figure 8 Egger’s regression asymmetry plots ( A. CAE-OR B. CAE-HR C. CAD D. ACS E. CVA F.PAOD G. HF H.Arrhythmia)

Except for the ACS , the results for every outcome could be considered as stable. In terms of the ACS, except for the (Torsten Dahlen,2016), omitting anyone of the studies would change the results.

| A  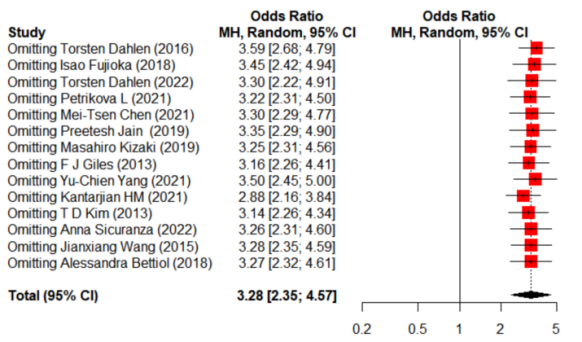 | B  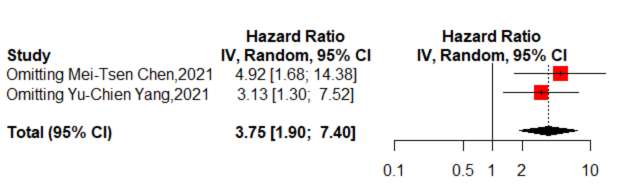 |
| --- | --- |
| C  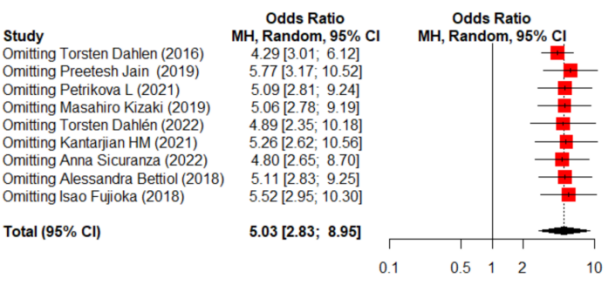 | D  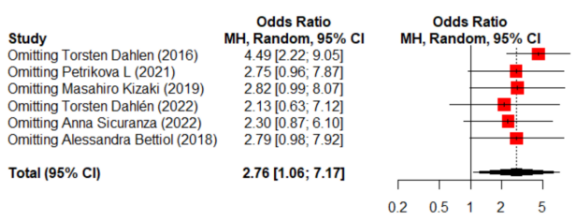 |
| E  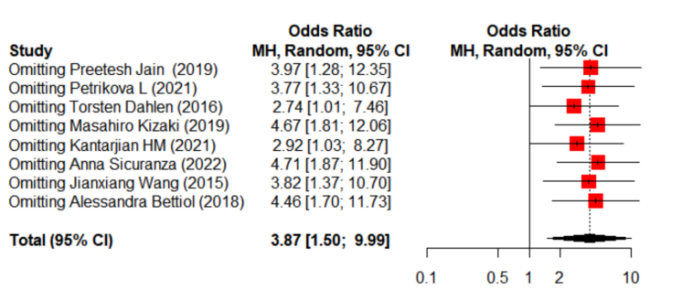 | F  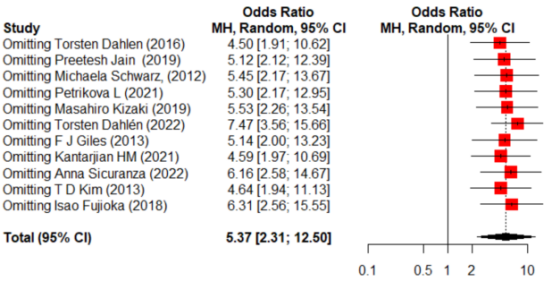 |
| G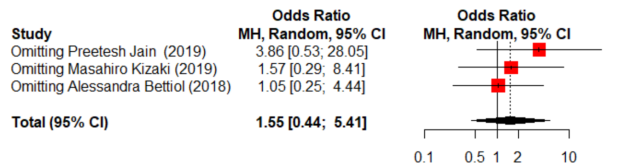 | H  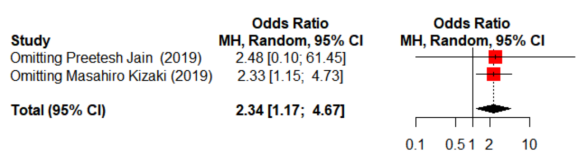 |

Figure 9 Sensitivity analysis ( A. CAE-OR B. CAE-HR C. CAD D. ACS E. CVA F.PAOD G. HF H.Arrhythmia)

(Torsten Dahlen,2016) contributed the most to heterogeneity in the comparison of CAD, ACS, CVA, while in CAE, PAOD and HF, (Yu-Chien Yang,2021), (Torsten Dahlén,2022)and (Alessandra Bettiol,2018) contributed the most to heterogeneity respectively.

| A  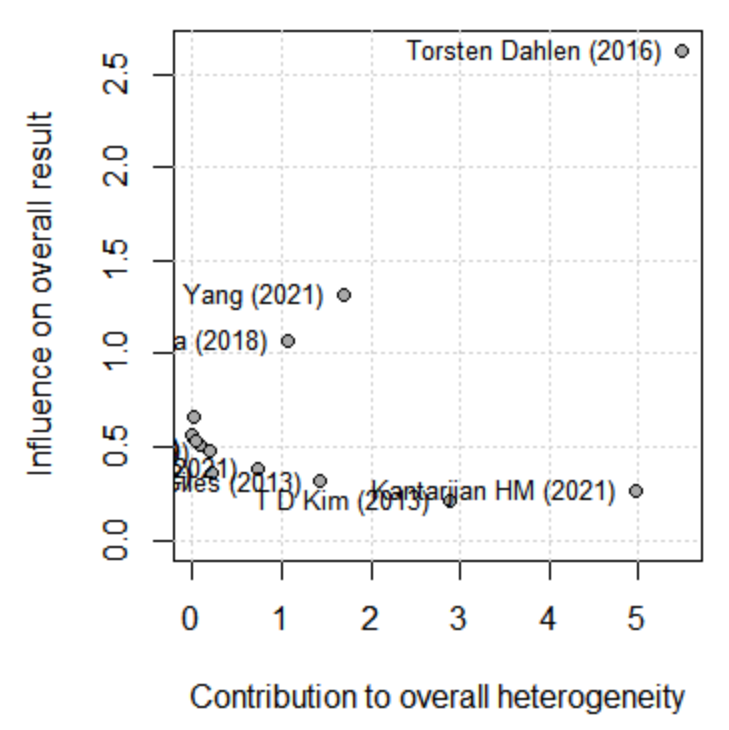 | B  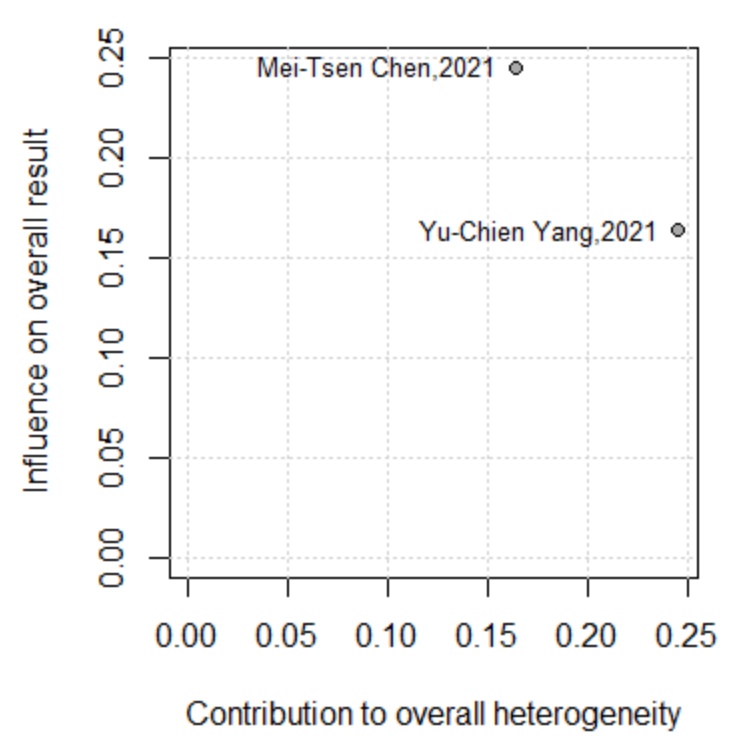 |
| --- | --- |
| C  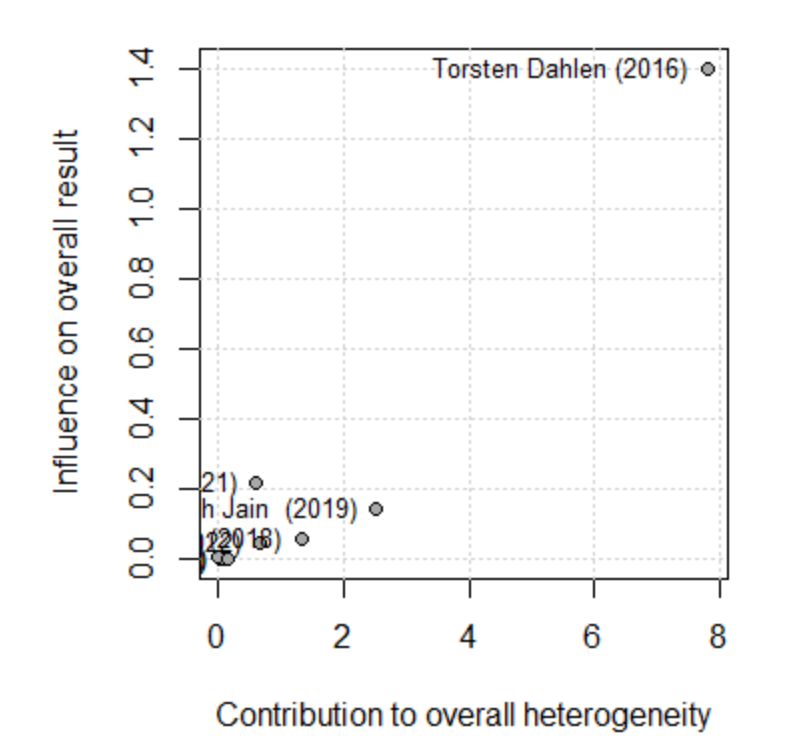 | D  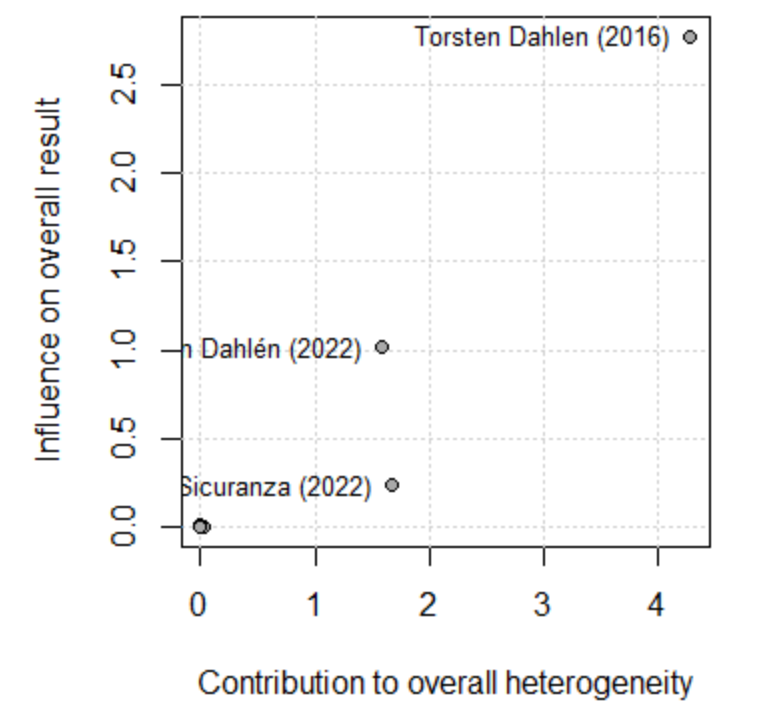 |
| E  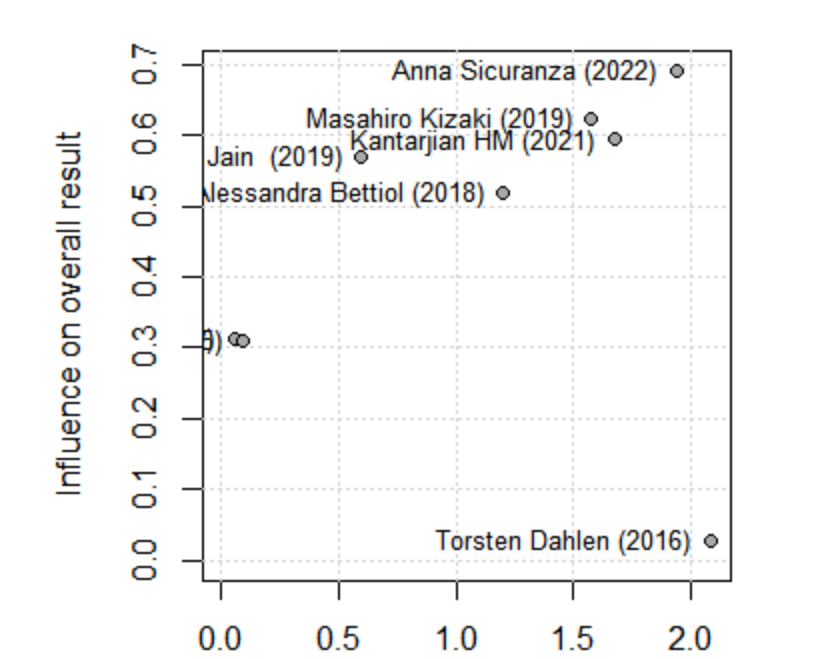 | F  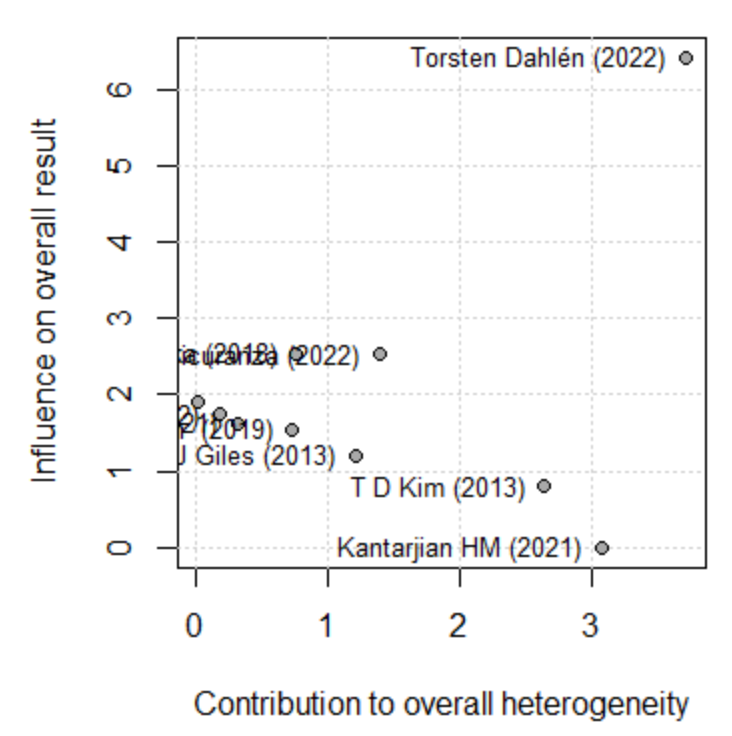 |
| G  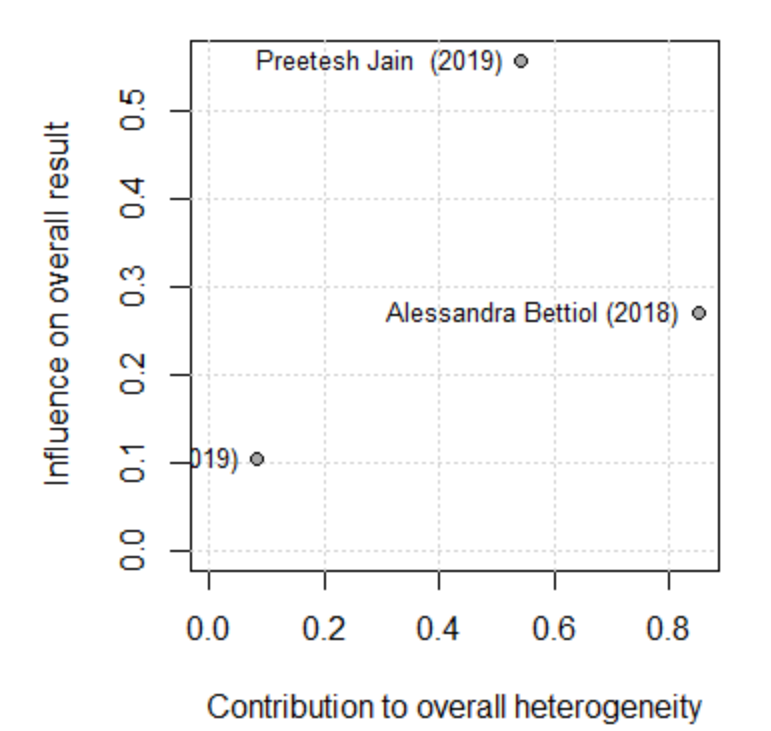 | H  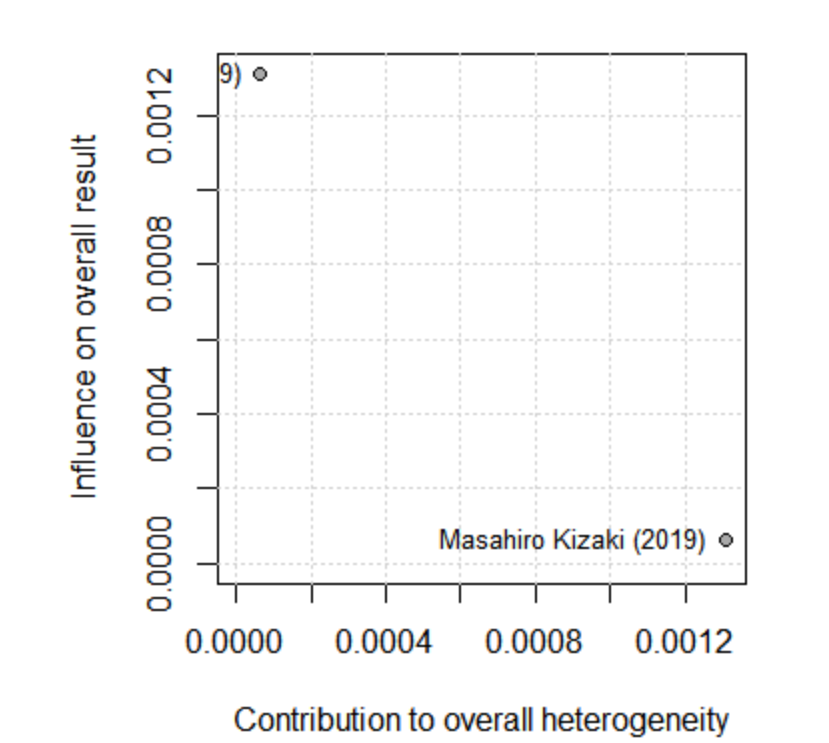 |

Figure 10 Baujat Plots for heterogeneity analysis( A. CAE-OR B. CAE-HR C. CAD D. ACS E. CVA F.PAOD G. HF H.Arrhythmia)


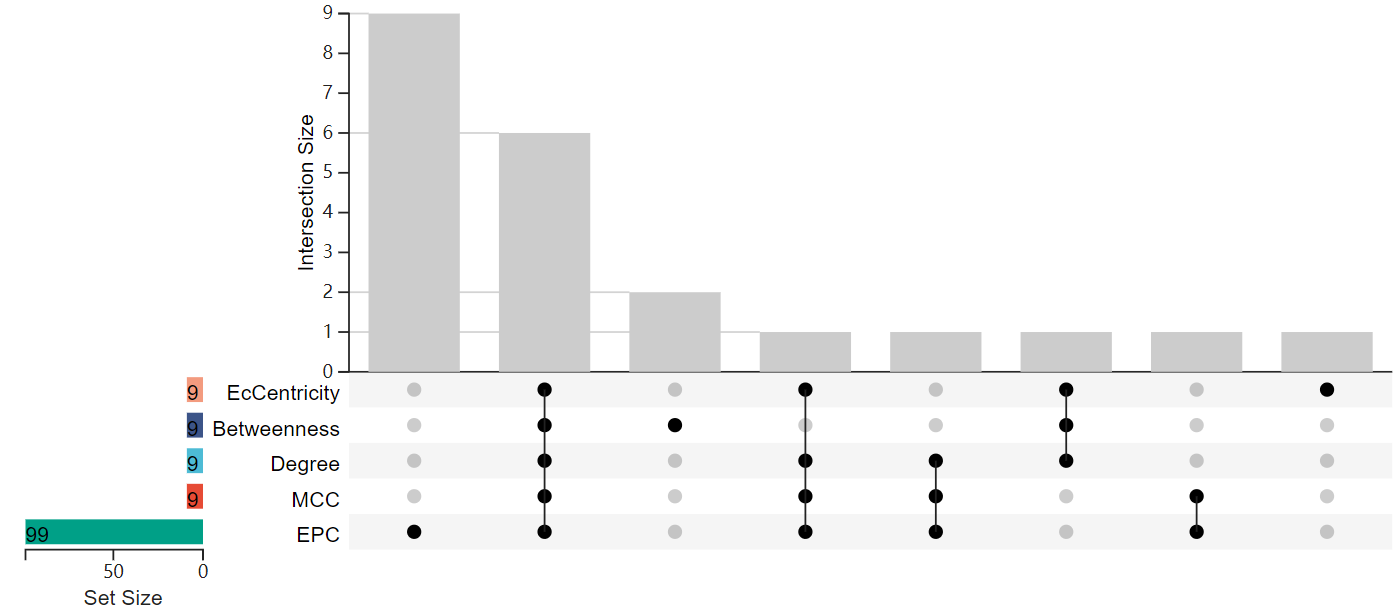


Figure 11 Upset-R plots for TOP 10 hub genes ( human cardiomyocytes)


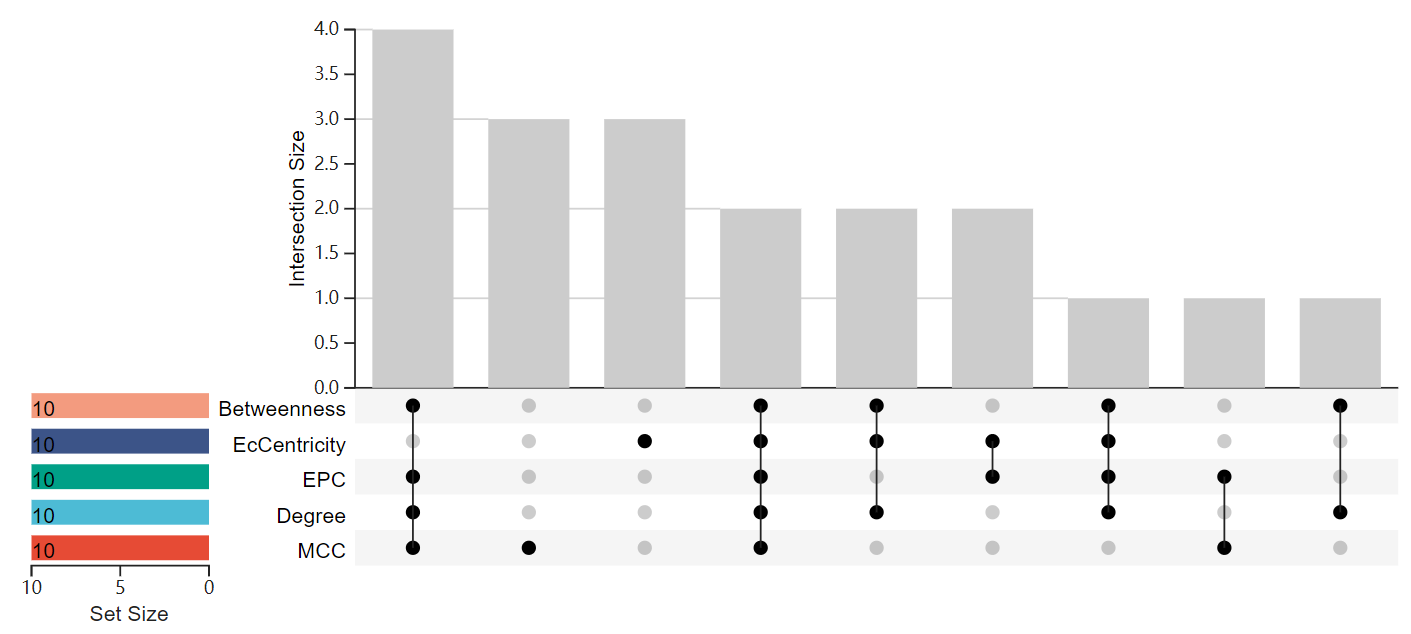


Figure 12 Upset-R plots for TOP 10 hub genes(Mus musculus liver samples)
